# Trapping of different stages of BaTiO_3_ reduction with LiH†

**DOI:** 10.1039/d0ra07276a

**Published:** 2020-09-24

**Authors:** Hua Guo, Aleksander Jaworski, Zili Ma, Adam Slabon, Zoltan Bacsik, Reji Nedumkandathil, Ulrich Häussermann

**Affiliations:** Department of Materials and Environmental Chemistry, Stockholm University SE-10691 Stockholm Sweden ulrich.haussermann@mmk.su.se; Institute of Inorganic Chemistry, RWTH Aachen University Landoltweg 1 DE-52074 Aachen Germany

## Abstract

We investigated the hydride reduction of tetragonal BaTiO_3_ using LiH. The reactions employed molar H : BaTiO_3_ ratios of 1.2, 3, and 10 and variable temperatures up to 700 °C. The air-stable reduced products were characterized by powder X-ray diffraction (PXRD), scanning electron microscopy, thermogravimetric analysis (TGA), X-ray fluorescence (XRF), and ^1^H magic-angle spinning (MAS) NMR spectroscopy. Effective reduction, as indicated by the formation of dark blue to black colored, cubic-phased, products was observed at temperatures as low as 300 °C. The product obtained at 300 °C corresponded to oxyhydride BaTiO_∼2.9_H_∼0.1_, whereas reduction at higher temperatures resulted in simultaneous O defect formation, BaTiO_2.9−*x*_H_0.1_□_*x*_, and eventually – at temperatures above 450 °C – to samples void of hydridic H. Concomitantly, the particles of samples reduced at high temperatures (500–600 °C) display substantial surface alteration, which is interpreted as the formation of a TiO_*x*_(OH)_*y*_ shell, and sintering. Diffuse reflectance UV-VIS spectroscopy shows broad absorption in the VIS-NIR region, which is indicative of the presence of n-type free charge carriers. The size of the intrinsic band gap (∼3.2 eV) appears only slightly altered. Mott–Schottky measurements confirm the n-type conductivity and reveal shifts of the conduction band edge in the LiH reduced samples. Thus LiH appears as a versatile reagent to produce various distinct forms of reduced BaTiO_3_ with tailored electronic properties.

## Abstract 2

Different forms of reduced BaTiO_3_, which include oxyhydride BaTiO_2.9_H_0.1_ and O-deficient BaTiO_2.9−*x*_H_0.1_□_*x*_, were obtained from reactions with LiH at various temperatures.

## Graphical abstract

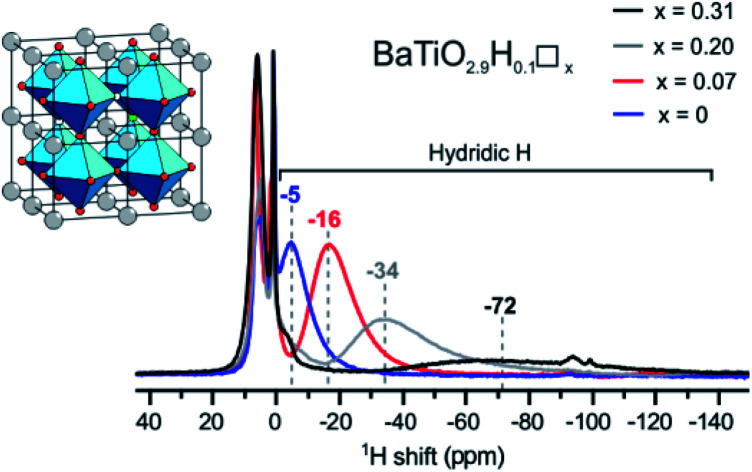

## 1 Introduction

**Fig. 1:**
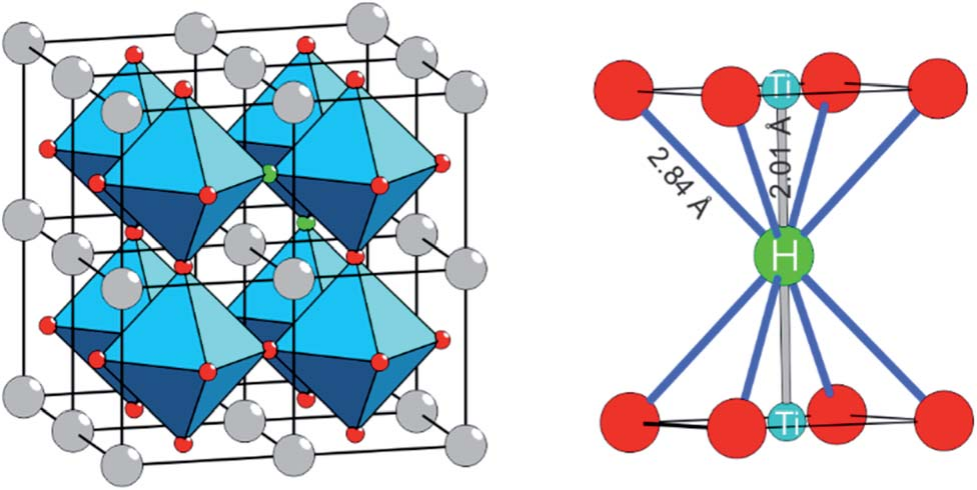
The structure of BaTiO_2.75_H_0.25_ represented as 2 × 2 × 2 supercell of cubic BaTiO_3_ in which two O atoms (red circles) were randomly replaced by two H atoms (green circles). The right hand side shows the local coordination of H in BaTiO_3−*x*_H_*x*_. The Ti is in a mixed IV/III oxidation state.

The discovery that the archetypical perovskite BaTiO_3_ can be converted to BaTiO_3−*x*_H_*x*_ (with H-contents up to *x* ≈ 0.6) by hydride reduction sparked enormous interest.^1–3^ The oxyhydride BaTiO_3−*x*_H_*x*_ attains a cubic structure in which O^2−^ and H^−^ ions commonly – and as in a solid-solution – form an octahedral environment around Ti that is now in a mixed IV/III oxidation state (Fig. 1).^1^ The material is stable in air and water. Further, BaTiO_3−*x*_H_*x*_ is stable at elevated temperatures of up to approximately 400 °C above which hydrogen is released. When present, oxygen is scavenged and BaTiO_3_ is retained. In inert gas atmospheres containing D_2_ a hydride exchange H/D occurs at hydrogen release temperatures.^4^ Also, oxynitrides BaTiO_3−*x*_N_*y*_ may be prepared by heating BaTiO_3−*x*_H_*x*_ under N_2_ flow at 400–600 °C.^5^ These observations led to the conclusion that the hydride species in BaTiO_3−*x*_H_*x*_ is labile and that the material represents a versatile precursor toward new mixed-anion compounds.^6^ Lately it has been shown that BaTiO_3−*x*_H_*x*_ also shows a remarkable activity as heterogeneous catalyst for ammonia synthesis, possibly following the Mars–van Krevelen mechanism.^7^

Hydride reduction of BaTiO_3_ involves the reaction of pelletized mixtures of perovskite with a metal hydride, typically CaH_2_, at elevated temperatures (550–600 °C) over prolonged periods of time (several days–week) in closed ampules.^1,2^ The mechanism or processes behind the metal hydride reduction of BaTiO_3_ is not well investigated and far from understood. Reaction conditions can be varied by the choice of reducing agent (*i.e.* metal hydride), reaction temperature and time, and the activity/concentration of H^−^. The active reducing species may be H^−^ or H_2_, or a combination.^8,9^ Typically, in the hydride reduction of transition metal oxides, the reduction is accompanied with O removal (*i.e.* O vacancy formation), and frequently there is a strict topotactic relationship between the original oxide and its reduced form.^10,11^ Actually, only in rare cases transition metal reduction and hydride ion insertion occur simultaneously, leading to oxyhydrides.^12^

An important question is what factors govern oxyhydride formation as opposed to exclusive vacancy formation during hydride reduction. What mechanism could apply for BaTiO_3_ reduction? We recently showed that the reduction of BaTiO_3_ can be achieved with a larger range of metal hydrides.^13^ In contrast to previous studies we found that hydride reduction leads to phases BaTiO_3−*x*_H_*y*_□_(*x*−*y*)_ with comparatively low H content and, accordingly, large O vacancy concentrations. Here we report a study using LiH, which revealed several peculiarities. First with LiH the reduction of BaTiO_3_ can be performed at extraordinarily low temperatures, 300 °C, which yields oxyhydride BaTiO_2.9_H_0.1_. At higher temperatures, O vacancies are introduced at the same time while the hydridic H-concentration appears unchanged. At yet higher temperature (500–600 °C) hydridic H is absent in reduced samples and particles show substantial surface alteration, which is interpreted as the formation of a TiO_*x*_(OH)_*y*_ shell. Thus LiH appears as a versatile reagent to produce various distinct forms of reduced BaTiO_3_.

## 2 Experiments

### 2.1 Synthesis

As starting materials we used BaTiO_3_ (500 nm particle size, 99.9% purity, ABCR GmbH) and LiH powder (95%, Sigma Aldrich). Prior to use, BaTiO_3_ was dried overnight in an oven at 200 °C. All steps of sample preparation for the synthesis reactions were performed in an Ar filled glove box. For a typical synthesis ∼0.5 g (2.1 mmol) of BaTiO_3_ was intimately mixed with LiH by grinding the materials together in an agate mortar for 20 min. We considered the molar proportions BaTiO_3_ + *n*LiH, with *n* = 1.2, 3, and 10. The BaTiO_3_/LiH mixture was subsequently pressed into a pellet with a diameter 9 mm. The pellet was then sealed inside a stainless steel ampule (with dimensions 10 mm ID and 50 mm length), which – after removal from the glove box – was placed in a box furnace. A K-type thermocouple was inserted and located in close proximity to the metal ampule. Ampules were heated for typically 48 h. Reaction temperatures varied from 250 to 700 °C. After cooling to room temperature, ampules were opened and the products washed with 0.1 M acetic acid (HAc) solution to remove excess LiH and metal oxide/hydroxide formed during hydride reduction. For washing, the pellets were crushed and sonicated with 50 mL HAc and then centrifuged. The procedure was repeated 3 times. As a last step, products were treated with 20 mL 95% ethanol and then dried at 120 °C under dynamic vacuum (<10^−5^ bar). The dried products corresponded to dark blue powders.

### 2.2 Powder X-ray diffraction (PXRD) analysis

PXRD patterns were collected on a Panalytical X'Pert PRO diffractometer operated with Cu Kα radiation and in *θ*–2*θ* diffraction geometry. Powder samples were mounted on a Si wafer zero-background holder and diffraction patterns measured in a 2*θ* range of 20°–120° with 0.016° step size. The contribution of Kα_2_ radiation to the PXRD patterns was removed using the Panalytical X'Pert HighScore Plus software. The Rietveld method as implemented in the FullProf program was used for structure and phase analysis.^14^ A six-coefficient polynomial function was applied for the background. The peak shape was described by a pseudo-Voigt function. Patterns with pronounced peak shape asymmetries were refined as mixtures of two cubic phases. Site occupancies for the O atoms could not be refined reliably and were constrained to 1.

### 2.3 Scanning electron microscopy (SEM) investigations

SEM investigations were carried out using a JEOL JSM-7000F machine equipped with a Schottky-type field emission gun and energy and wavelength dispersive detectors from Oxford instruments. Images were recorded in backscattering mode with an accelerating voltage of 15 kV. Samples for imaging were prepared by first producing a homogeneous suspension of particles in ethanol by sonication. Then droplets of the suspension were applied onto surface-polished aluminum pin stubs and left to dry. Energy-dispersion X-ray (EDS) and wave-dispersion X-ray (WDX) data were collected with an accelerating voltage of 8 kV and processed with the INCA software. Samples for EDS and WDX analysis were prepared by spreading sample powders on copper grids. Measurements were carried out by focusing the electron beam on selected individual particles and then collect statistical results. Quantification optimization was done with respect to BaTiO_3_ starting material.

### 2.4 X-ray fluorescence spectrometry (XRF) analysis

XRF was performed on a PANalytical Epsilon3 spectrometer, with working condition of 20 kV, high-resolution detector SSD, medium of air and filter of Al-200. Powder samples were pressed into pellets with diameter of 8 mm. Quantification results were calculated by comparing to reference standards (mixtures of BaTiO_3_ and TiO_2_ with known ratio).

### 2.5 Thermogravimetric analysis (TGA)

TGA experiments were carried out using a TA instruments Discovery system. The samples (∼15 mg powders) were heated in a platinum crucible from room temperature to 900 °C with a heating rate of 5 °C min^−1^. A dry air gas flow of 20 mL min^−1^ was applied.

### 2.6 UV-VIS diffuse reflectance spectroscopy

UV-VIS diffuse reflectance measurements were performed at room temperature on finely ground samples with particle size < 300 nm. Spectra were recorded in the range from 200 to 800 nm with an Agilent Cary 5000 UV-VIS-NIR spectrometer equipped with a diffuse reflectance accessory from Harrick. A polytetrafluoroethylene (PTFE) pellet (100% reflectance) was used as the reference.

### 2.7 Magic-angle spinning (MAS) NMR spectroscopy

The ^1^H and ^7^Li MAS NMR experiments were performed at a magnetic field of 14.1 T (600.12 and 233.23 MHz Larmor frequencies for ^1^H and ^7^Li, respectively) and MAS frequency of 60 kHz on a Bruker Avance-III spectrometer equipped with a 1.3 mm MAS HX probe. Proton spectra were acquired using a rotor-synchronized, double-adiabatic spin-echo sequence with a 90° excitation pulse of 1.2 μs followed by a pair of 50 μs tanh/tan short high-power adiabatic pulses (SHAPs) with 5 MHz frequency sweep.^15–17^ All pulses were applied at a nutation frequency of 208 kHz. 4096 signal transients with a 5 s recycle delay were accumulated for each sample. Shifts were referenced with respect to tetramethylsilane (TMS) at 0 ppm. ^7^Li spectra were collected using single-pulse (“Bloch decay”) protocol with 90° excitation pulse of 1.40 μs (179 kHz nutation frequency), 60 s recycle delay, and 64 signal transients per sample. Shifts were referenced with respect to solid LiF at 0 ppm. The amount of Li in each sample was estimated by relating its integrated ^7^Li NMR signal intensity to that of LiF in the same rotor volume, assuming molecular mass and density of 233.192 g mol^−1^ and 6.02 g cm^−3^ for BaTiO_3_ and 25.939 g mol^−1^ and 2.64 g cm^−3^ for LiF, respectively.

### 2.8 Mott–Schottky measurements

Powder samples were assembled into particle-base thin film electrodes on fluorine-doped tin oxide (FTO) glass substrate *via* electrophoretic deposition (EPD) process. Briefly, a homogeneous suspension was prepared by mixing 30 mg powder samples and 10 mg iodine in 30 mL of acetone *via* sonication. Two pre-cleaned FTO slides were immersed in the above suspension with 1 cm distance, and a DC potential of 30 V was applied between them for 3 min. After EPD process, the FTO slides with thin film were washed with water and dried naturally. 0.1 M NaOH (pH 13), in which oxyhydrides are stable,^1^ was used as electrolyte to perform Mott–Schottky measurements. The measurements were conducted in the conventional three-electrode system where a platinum wire, a 1 M Ag/AgCl electrode and the thin film on FTO served as counter, reference electrode and working electrode, respectively. The potentials were recorded *versus* 1 M Ag/AgCl and converted *versus* reversible hydrogen electrode (RHE) according to *E*_RHE_ (V) = *E*_Ag/AgCl_ + (0.059 × pH) + *E*^θ^_Ag/AgCl_. The measurements were carried out by using the Gamry INTERFACE 1010T Potentiostat/Galvanostat/ZRA workstation in a dark setting at ac amplitude of 5 mV and a frequency of 10 Hz.

## 3 Results and discussion

### 3.1 Hydride reduction of BaTiO3 with 1.2 M LiH

**Fig. 2:**
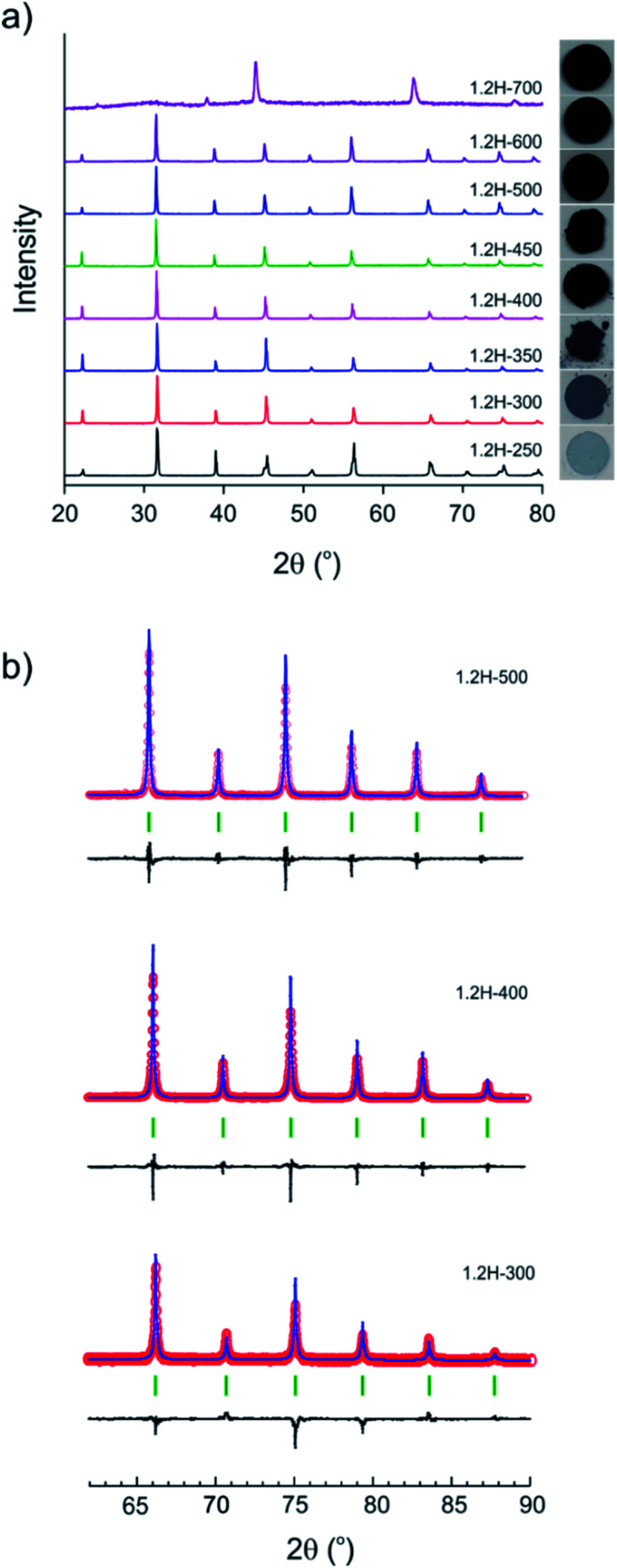
(a) PXRD patterns of products obtained from reactions with 1.2 M LiH at various temperatures during 48 h. The visual appearance/color of each sample is shown in the right panel. (b) Sections of Rietveld plots for the PXRD patterns of samples obtained at 300, 400, and 500 °C.

The evolution of products with increasing temperature, from 250 to 700 °C, during 2 day experiments is displayed in Fig. 2. Table 1 contains the results from the refinement of the PXRD patterns. (Reduced samples are referred to as 1.2H-xxx where xxx denotes the synthesis temperature in °C.) At 250 °C the product had a pale blue color and remained tetragonal. At higher temperatures, from 300 to 600 °C, reduced samples were cubic and showed a dark blue to black color. The lattice parameter increases slightly from 4.005 Å for 1.2H-300 to 4.021 Å for 1.2H-450 and then remains more or less constant for the products obtained at higher temperatures. According to PXRD, the cubic products obtained at 300–600 °C show high crystallinity and appear to be single phase. A broadening of reflections, indicating reduced particle size and/or increased strain, or an increased amorphous background, indicating a diminished crystallinity is not apparent (Fig. 2b). The refinement details are given in the ESI section (Table S1 and Fig. S1).† The product obtained at 700 °C was found to be largely amorphous. The reflections of the crystalline component could be indexed to a F-centered cubic lattice.

**Table 1:** Characterization of synthesis products from reactions with 1.2 M LiH during 2 day experiments at various temperatures

Reaction temperature (°C)	Lattice parameters (Å)	Volume (Å^3^)	*X*_TG, H_ a	*X*_TG, □_ b	*X*_NMR, H_	*X*_□_	Molar Ba/Ti ratio (XRF)
250	*a* = 3.9955 (1)	64.250 (4)	0.01	0.01			
	*c* = 4.0247 (2)						
300	4.0046 (1)	64.219 (5)	0.08	0.07	0.09	0	
350	4.0069 (2)	64.329 (5)	0.16	0.15	0.09	0.07	0.99
375	4.0084 (1)	64.405 (4)	0.19	0.18			
400	4.0122 (1)	64.588 (4)	0.29	0.27	0.09	0.20	0.98
425	4.0156 (1)	64.754 (3)	0.32	0.30			
450	4.0212 (1)	65.021 (3)	0.40	0.37	0.09	0.31	0.93
500	4.0212 (1)	65.022 (3)	0.40	0.38	—	0.38	0.82
600	4.0200 (1)	64.965 (3)	0.08	0.07			0.60
700	Essentially amorphous						

a *X*_TG, H_ refers to a reaction BaTiO_3−*x*_H_*x*_ + 0.75*x*O_2_ → BaTiO_3_ + 0.5*x*H_2_O.

b *X*_TG, □_ refers to a reaction BaTiO_3−*x*_ + 0.5*x*O_2_ → BaTiO_3_.

**Fig. 3:**
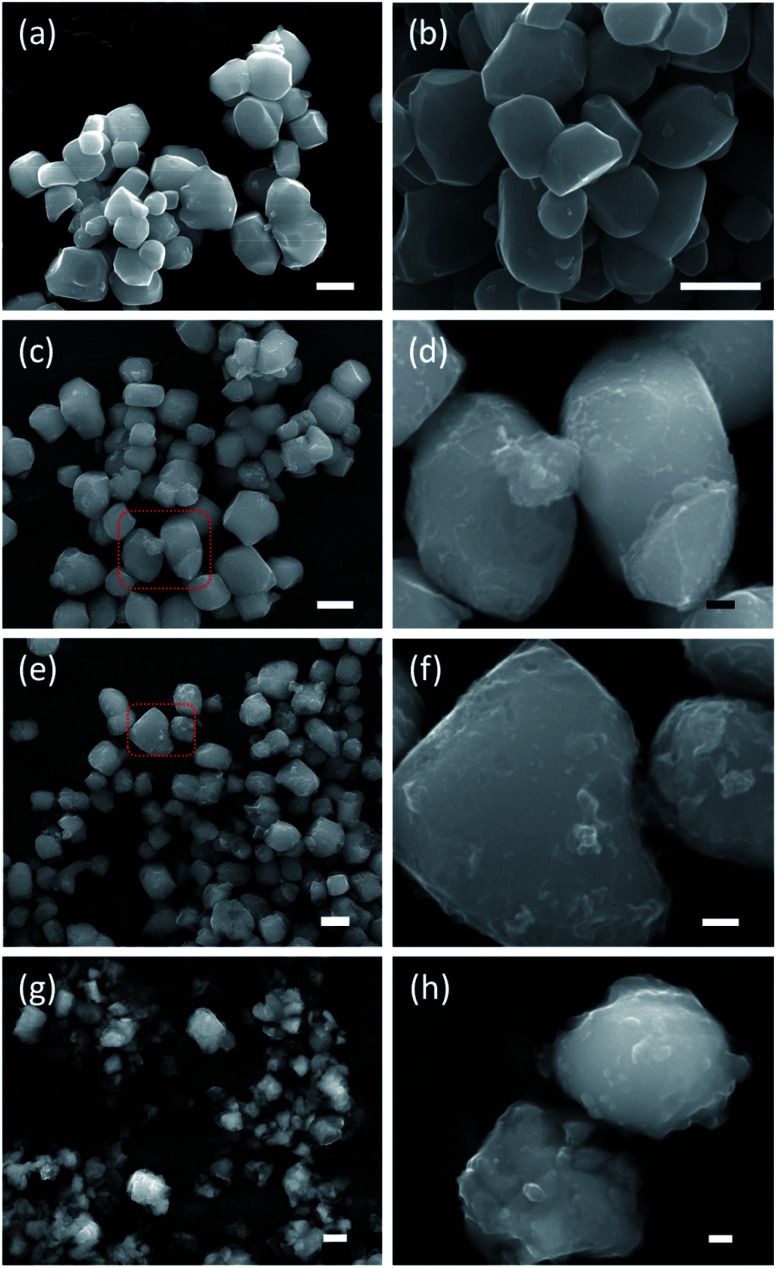
SEM images of the BaTiO_3_ starting material (a) and 1.2 M LiH reduced samples at 350 °C (b), at 450 °C (c) and (d), at 500 °C (e) and (f), and at 600 °C (g) and (h). The scale bar is 500 nm for (a)–(c), (e), (g) and 100 nm for (d), (f), (h).

Fig. 3 shows SEM images of the starting material and selected reduced samples. The average particle size of the starting material, tetragonal BaTiO_3_, was specified by the manufacturer as 0.5 micron. This is confirmed in Fig. 3a, although some particle size distribution is noticeable. The particle morphology of the starting material is retained in the products obtained at 300, 350, and 400 °C. This is also largely the case for 1.2H-450 and 1.2–500. However, it is noticeable that the particles of 1.2H-450 attained a roughened surface and the ones of 1.2H-500 display severe surface alteration, which is accompanied by onset of particle sintering. The product obtained at 600 °C appears then significantly changed, despite its cubic PXRD pattern. Here the original morphology of the BaTiO_3_ particles is not recognizable anymore. 1.2H-600 particles seem smaller, rounded, and especially largely sintered. 1.2H-700 consists of micron-long needles – which are attributed to crystalline component – and irregular fused particles, which are attributed to amorphous component (see ESI, Fig. S4†). We will exclude 1.2H-700 from further discussion in this manuscript.

Thermogravimetric analysis has been frequently used to assess the H content and/or O defect concentration of reduced BaTiO_3_.^1,2,13^ TGA under flowing air monitors the reactions

1 BaTiO_3−*x*_H_*x*_ + 0.75*x*O_2_ → BaTiO_3_ + 0.5*x*H_2_O

and

2 BaTiO_3−*x*_ + 0.5*x*O_2_ → BaTiO_3_.

**Fig. 4:**
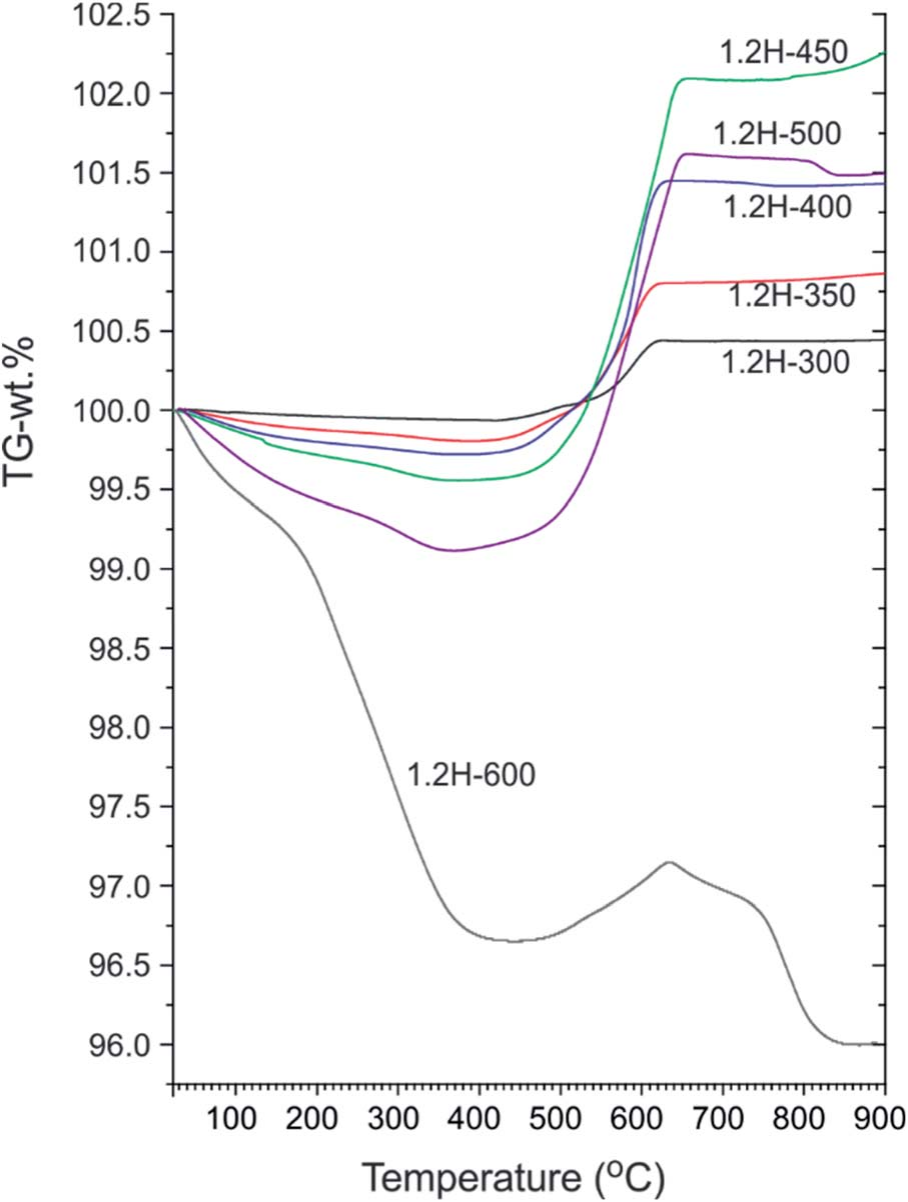
TGA traces of products obtained from 1.2 M LiH reduction during 48 h at different temperatures.

Fig. 4 shows a compilation of TGA traces for 1.2 M LiH-reduced samples. All samples show initially a weight loss which is attributed to loss of surface hydroxyl and secondary water.^18,19^ For 1.2H-300 this weight loss is minute (0.1–0.15%) and very similar to that of pristine starting material (which shows a continuous weight loss amounting to 0.15% up to 700 °C).^13^ For 1.2H-450 the initial weight loss is increased to 0.45% and for 1.2H-500 it is substantial, 0.9% at 350 °C. Obviously, compared with reduction at lower temperatures, the concentration of surface hydroxyl/water is largely increased for 1.2H-450, and especially for 1.2H-500. The initial weight loss seen in TGA most likely correlates with the surface alteration of 1.2H-450 and 1.2H-500 particles seen in SEM. This suggests that LiH reduction at temperatures higher than 400 °C increasingly affects and modifies the surface of BaTiO_3_ particles, which, after washing, results in a higher concentration of hydroxyl and adsorbed water.

Characteristic for all products obtained at synthesis temperatures up to 500 °C is a steep subsequent weight increase above 450 °C. This should be due to oxidation, which for these samples is completed above 700 °C. The associated *x* values according to eqn (1) and (2) are contained in Table 1. These values range from 0.08 (1.2H-300) to 0.36 (1.2H-500) and thus appear rather modest with respect to earlier values reported for hydride reduced BaTiO_3_ (*x* = 0.5–0.6).^1,2^ The behavior of 1.2H-600 is then radically different. Its TG trace shows a large, ∼3%, weight loss up to 400 °C, then some small weight gain (0.5%), and finally further weight-loss above ∼650 °C up to about 800 °C. The product after the TG/air treatment (*i.e.* after heating and cooling to 900 °C) is white and corresponds to tetragonal BaTiO_3_ for the samples 1.2H-300 to 1.2H-450 (see ESI, Fig. S5 and S6†). The pattern of TG/air treated 1.2H-500 contains traces of BaTi_5_O_11_. The pattern of oxidized 1.2H-600 shows clearly the presence of BaTi_5_O_11_ and in addition traces of Li_0.67_Ba_2_Ti_5.33_O_13_, which indicates Li incorporation in the original 1.2H-600 sample (see ESI, Fig. S5b†).

To summarize: LiH reduces BaTiO_3_ significantly already at very low temperatures, 300 °C. This is remarkable since previously employed reducing agents, such as CaH_2_, MgH_2_, NaAlH_4_ or NaBH_4_, are only effective at temperatures above 400 °C.^1,13^ Products from LiH reductions at 450–600 °C deviate from the expected (or previously observed) behavior of metal hydride reduced BaTiO_3_ because of their significant initial TGA weight loss (>0.3 wt%). This can only be explained by water loss and signals significant surface alteration of reduced BaTiO_3_ particles. Next, we address in more detail the composition of the LiH reduced samples, in particular H-incorporation *vs.* O deficiency, and the possibility of Li incorporation.

### 3.2 Compositional analysis of 1.2 M LiH reduced samples

We have used ^1^H MAS NMR earlier for the analysis of metal hydride reduced BaTiO_3_ samples.^13^^1^H MAS NMR is especially useful for unambiguously determining the hydridic H content, which then allows the deduction of O defect concentrations from TGA measurements. Importantly, protic H from (surface) hydroxyl and hydridic H as part of the bulk structure can be discriminated as positive and negative chemical shift contributions, respectively, in the spectra.^13^

**Fig. 5:**
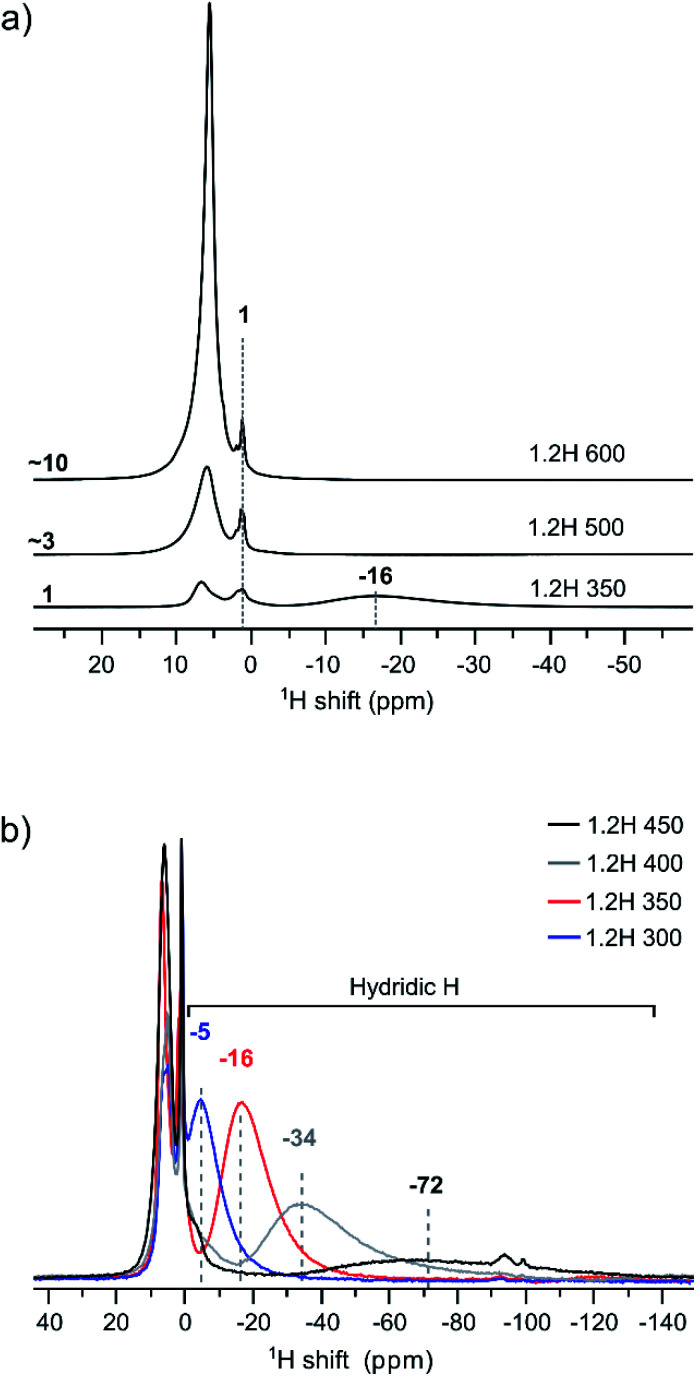
(a) ^1^H MAS NMR spectra of products obtained from 1.2 M LiH reduction during 48 h at different temperatures. The increase of protic signal (positive ppm) relative to 1.2H-350 is indicated. (b) Comparison of spectra emphasizing the negative ppm range with the hydridic H signal.

Fig. 5 compiles the ^1^H NMR spectra for the 1.2H-300, -350, -400, -450, 500, and -600 samples. Hydric H expresses itself as a single broad resonance peak at negative chemical shift whereas protic resonances at ∼1 ppm and in the region 6–7 ppm are attributed to surface OH species and secondary water.^13^ The increase of the protic H concentration, in particular the ones from surface OH, with increasing synthesis temperature of samples is highlighted in Fig. 5a. The relative increase of protic H signal (in the positive ppm range) for 1.2H-500 and 1.2H-600 with respect to 1.2H-350 is ∼3 and ∼10. This correlates roughly with the relative weight losses seen up to 400 °C in the TG experiments (*i.e.* 0.2%–0.9%–3.3%, *cf.*Fig. 4). The hydridic resonance is seen at −16 ppm for 1.2H-350.

Fig. 5b shows the evolution of the hydridic resonance with increasing synthesis temperature of samples. We notice a consistent shift towards more negative ppm values: for 1.2H-300 it is at −5 ppm, for 1.2H-350 at −16, for 1.2H-400 at −34, and for 1.2H-450 at −72 ppm. At the same time there is a significant broadening of the signal. Interestingly, the integral of the deconvoluted negative ppm resonance is very similar across the samples, which indicates that very similar amounts of H were incorporated. We showed earlier that the amount of hydridic H in reduced samples can be quantified by relating the ^1^H NMR signal of the BaTiO_3_ starting material to that of adamantane (C_10_H_16_) in the same rotor volume and under identical experimental conditions.^13^ When applying the same procedure for the LiH reduced samples, we find *X*_H_ = 0.09 (1) for all samples. This result is included in Table 1, which summarizes the properties of 1.2 M LiH reduced samples. A resonance at negative ppm values could not be discerned in the spectra of the 1.2H-500 and -600 samples, indicating the absence of hydridic H in samples that are prepared at temperatures above 450 °C.

With the knowledge of the hydridic H concentration, the corresponding weight increase according to eqn (1) can be calculated. The difference to the actual weight increase in the TGA experiment is then attributed to the simultaneous presence of O vacancies, *X*_□_, see Table 1. This suggests that LiH reduction produces three different materials with increasing annealing temperature: the 1.2H-300 sample corresponds to oxyhydride (with a rather low H content, BaTiO_2.9_H_0.1_), whereas the 1.2H-350, -400 and -450 samples show an increasing O defect concentration (while keeping the H content constant). 1.2H-500 is void of hydridic H and one would assume that this sample corresponds to O-deficient BaTiO_2.6_. At the same time we remember that, starting off 1.2H-450, LiH reduced samples show surface alteration which then culminates in particle sintering for 1.2H-600.

To analyze the compositional integrity of the 1.2 M LiH reduced samples we performed ^7^Li MAS NMR experiments. The spectra from the 1.2H-500 and -600 samples show that 1.2H-500 does not contain Li whereas a ^7^Li resonance is present in the spectrum of 1.2H-600, indicating some Li incorporation (see Fig. S7, ESI†). This is in agreement with the trace amount of Li_0.67_Ba_2_Ti_5.33_O_13_ identified in the PXRD pattern of 1.2H-600 after TG/air analysis. XRF analysis showed that the Ba/Ti ratio of 1.2 M LiH reduced samples is essentially 1 for 1.2H-300, -350, -400, but deviates significantly for 1.2H-450 (0.93) (*cf.* ESI, Fig. S8†). Samples which were reduced at even higher temperatures appear pronouncedly Ba deficient, which could be confirmed by WDX analysis, as shown in the ESI (Fig. S9).† This suggests that only samples reduced at 300–400 °C maintain their compositional integrity with respect to Ba/Ti and that with the onset of visible surface alteration (*i.e.* 1.2H-450) the metal composition of particles is changed.

**Fig. 6:**
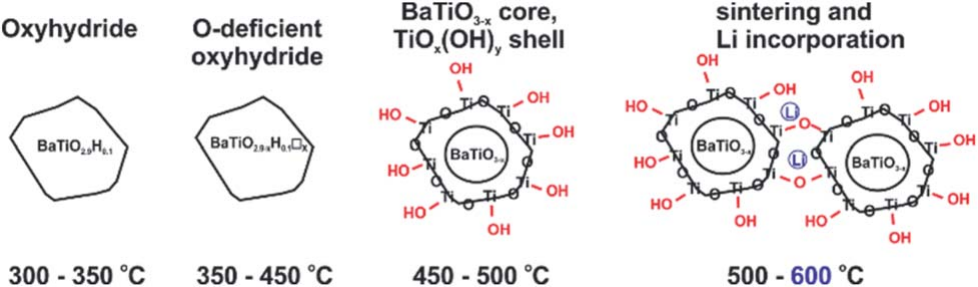
Evolution of products from LiH reduction of BaTiO_3_ with increasing synthesis temperature.

We conjecture that reduction at temperatures of 450 °C and higher – leading to surface modification – imply at the same time that the surface of particles is depleted of Ba. Upon washing, expelled BaO is removed whereas the surface converts to TiO_*x*_(OH)_*y*_. This is in agreement with the increasingly higher concentration of surface hydroxyl observed with increasing synthesis temperature. The 1.2H-500 sample would then correspond to “core–shell” particles with a BaTiO_3−*x*_ core and a TiO_*x*_(OH)_*y*_ shell/surface (with a volume ratio 5 : 1, according to XRF). The presence of a TiO_*x*_(OH)_*y*_ shell/surface could then also explain the occurrence of sintering as fusion of reduced BaTiO_3_ particles *via* condensation reactions of Ti coordinated surface hydroxyl. As shown by NMR, Li is incorporated in the 1.2H-600 sample. We believe that Li is part of Li–Ti–O phases and concentrated in the interface of fused particles. The Ba/Ti ratio for 1.2H-600 according to XRF is just 0.6, which would correspond to a BaTiO_3_(core)/TiO_2_(shell) ratio of 1.5 : 1. It is then surprising that 1.2H-600 maintains an average cubic perovskite structure and that the large volume fraction of compositionally different surface appears virtually invisible in the PXRD pattern (*cf.*Fig. 2 and S1i†). This may be explained by a severely disordered or even quasi-amorphous nature of the TiO_*x*_(OH)_*y*_ surface, similar to the surface disorder that was recently reported for tetragonal BaTiO_3_ nanocrystals.^20^Fig. 6 summarizes the conjectured evolution of products with increasing reduction temperature. The PXRD pattern of 1.2H-600 after TG/air treatment shows the presence of BaTi_5_O_11_ – in agreement with a Ba deficient sample – however, the fraction appears very small (*cf.* Fig. S5†). Another unexplained – but probably related – feature is the substantial (∼1%) TG weight loss of 1.2H-600 above 650 °C, which may be due to volatile Ba–Li–Ti–O species (*cf.*Fig. 4, note that also the TG trace of 1.2H-500 indicates some weight loss at high temperatures).

We now return to the ^1^H MAS NMR spectra shown in Fig. 5b. These spectra indicate a correlation between the shifts and linewidths of the hydridic ^1^H resonances, which broaden from 1.2H-300 to 1.2H-450 and concurrently shift towards more negative ppm values. A similar observation has been made earlier when the increasing negative shift was induced by the kind of the reducing agent (NaBH_4_, CaH_2_, NaAlH_4_, MgH_2_).^13^ The observed negative shift for the hydridic ^1^H has been attributed to the contact hyperfine interaction between the 3d electrons of Ti(iii) and the ^1^H nucleus. This shift mechanism is operative in both metallic systems and in paramagnetic insulators.^21^ For metallic BaTiO_3*x*_H_*x*_ it has been suggested that the electrons are delocalized in a conduction band,^22^ as opposed to a localized polaron scenario, and the hyperfine interaction results in a Knight shift that depends on the density of states at the Fermi level.^23^ The magnitude of the Knight shift is known to increase with the concentration of charge carriers.^24^ Thus one would expect that the charge carrier concentration in our samples increases with increasing Knight shift, that is, from 1.2H-300 to 1.2H-450.

### 3.3 Effect of larger LiH concentrations

We also performed hydride reduction experiments with a larger LiH concentration, 3 M LiH and 10 M LiH (accordingly these samples are abbreviated as 3H-xxx and 10H-xxx, respectively). Table 2 summarizes the properties of obtained products. A compilation of SEM images, PXRD patterns, *etc* is provided as ESI (Table S1, Fig. S2, S3, S10–S12).†

**Table 2:** Characterization of synthesis products from reactions with 3 M and 10 M LiH during 2 day experiments at various temperatures

	Reaction temperature	Lattice parameters (Å)	Volume (Å^3^)	*X*_TG, H_	*X*_TG, □_	*X*_NMR, H_	*X*_□_	Molar Ba/Ti ratio (XRF)
3H	350	4.0090 (1)	64.433 (3)	0.19	0.18	0.09	0.09	0.98
3H	500	4.0326 (1)	65.577 (4)	0.42	0.39	—	0.39	0.55
3H	700	Amorphous						
10H	350	4.0107 (1)	64.516 (3)	0.20	0.19	0.09	0.10	0.98
10H	500	4.0338 (1)	65.636 (3)	0.22	0.21	—	0.21	0.50
10H	700	Amorphous						

**Fig. 7:**
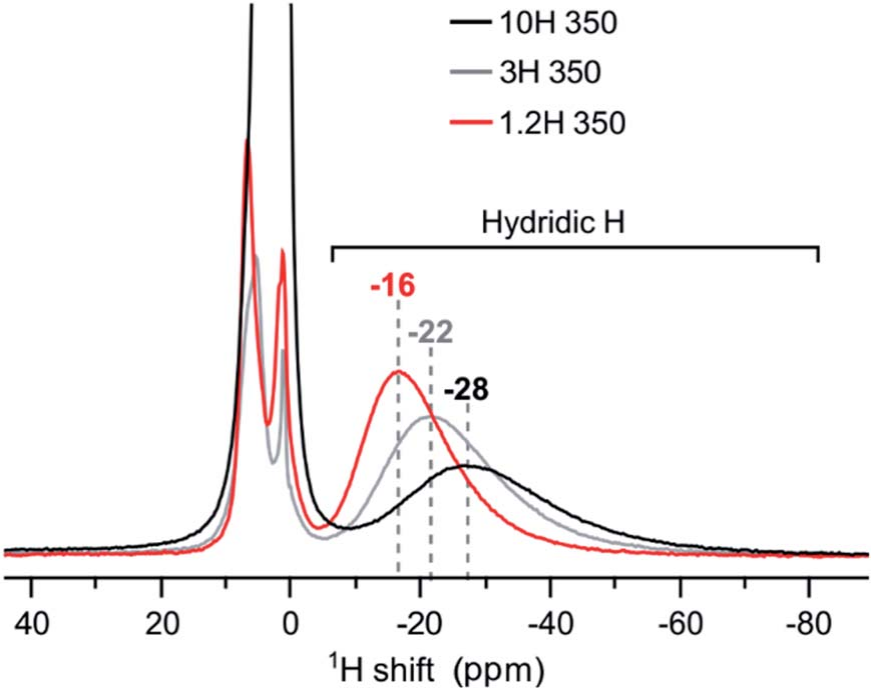
^1^H MAS NMR spectra of products obtained from reductions using variable LiH concentrations during 48 h at 350 °C.

At first sight, the products of 3H and 10H reductions at 350 and 500 °C appear similar to the corresponding 1.2H experiment. ^1^H NMR analysis revealed then slight but decisive differences. Fig. 7 shows the spectra for the samples obtained at 350 °C. The hydridic resonance, which is at −16 ppm for 1.2H-350 (*cf.*Fig. 6b), shifts to more negative ppm and concomitantly there is a broadening of the signal. Thus, increasing the LiH concentration has a similar effect as increasing the temperature during the reduction. Interestingly, the amount of incorporated, hydridic, H is not increased. The relative signal integrals of the negative ppm resonance are very similar for 1.2H-, 3H-, and 10H-350 and translate to *X* = 0.09 (1) for all samples. And analogous to the 1.2 M LiH reductions, the spectra for 3H-500 and 10H-500 do not exhibit a resonance in the negative ppm region, indicating the absence of hydridic H.

Earlier work has shown that hydride reduction of BaTiO_3_ depends strongly on the reducing agent (metal hydride) and that reduced BaTiO_3_ samples frequently represent complex, heterogeneous, materials, due to the simultaneous presence of vacancies and H in the anion substructure.^13^ With LiH it appears that a single reducing agent can produce in a controlled way different stages of reduced BaTiO_3_, depending on reaction temperature (*cf.*Fig. 6). Resonances of (hydridic) H substituting O in the cubic perovskite structure appear in the −5 to −70 ppm spectral region. The large range of negative chemical shifts and breadth of the signals signifies metallic conductivity and increasing structural disorder in the phase BaTiO_3−*x*_H_*y*_□_(*x*−*y*)_ which is obtained between 300 and 450 °C.

### 3.4 Spectroscopic and Mott–Schottky analysis

BaTiO_3_ is a wide gap semiconductor with a band gap energy of 3.2–3.4 eV.^25,26^ According to first principles calculations, the band gap is indirect for the tetragonal form and direct for the cubic (high temperature) polymorph.^27^ However, experimentally it appears to be difficult to assign the nature of the band gap and frequently a direct band gap is also assumed for the tetragonal form. BaTiO_3_ has attracted attention as photocatalyst and there have been numerous investigations into its electronic properties.^28,29^ For becoming useful photocatalysts nearly all wide gap semiconductors (TiO_2_, BiVO_4_, perovskites, *etc.*) require chemical modifications to generate donor/acceptor levels in the band gap, or noble metals to facilitate charge transfer and separation. Frequently, partial reduction can be used to improve photocatalytic and/or photo-electrochemical activity by “self-doping”. The most prominent example is the conversion of TiO_2_ to black TiO_2_, which has a blue color due to the absorption in visible and near-IR region.^30,31^ This is similar to hydride reduced BaTiO_3_ for which, however, electronic and photocatalytic properties are far less investigated. Originally BaTiO_3−*x*_H_*x*_, with *x* = 0.3 and 0.6 (as obtained from CaH_2_ reduction) has been attributed a high electronic conductivity.^1^ Later thin films of BaTiO_3−*x*_H_*x*_ were found to be semiconducting for *x* < 0.2 and metallic for 0.2 < *x* < 0.6.^3^

**Fig. 8:**
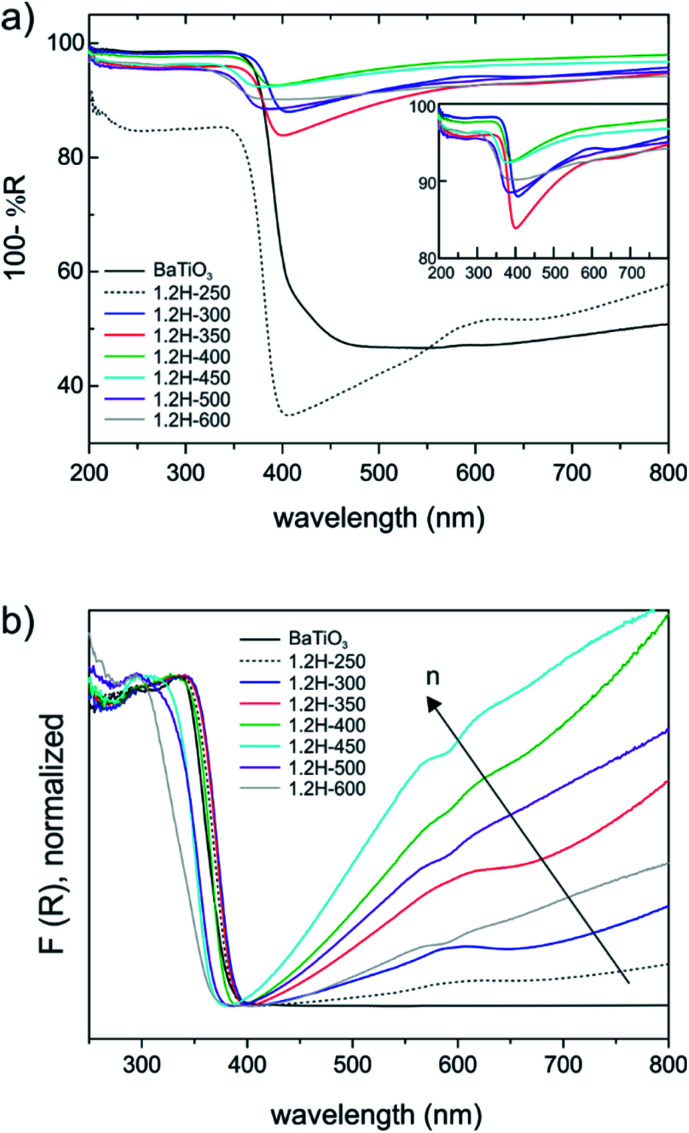
(a) Diffuse reflectance UV-VIS spectra of 1.2 M LiH reduced samples. The inset shows a close up of the samples 1.2H-300 to 1.2H-600. (b) Normalized Kubelka–Munk function of spectra comparing all samples. The arrow indicates increased charge carrier density.

UV-VIS diffuse reflectance spectra are shown in Fig. 8. The weakly reduced product obtained from reduction at 250 °C (1.2H-250) and the precursor (which both have the tetragonal structure) are different from the cubic products obtained at higher temperatures. The latter all exhibit a broad absorption in the visible and near-IR region (Fig. 8a). The actual absorption edge, corresponding to interband transitions, is around 400 nm, which relates well to the band gap energy. An important observation is that the near IR-VIS absorption upon approaching the upturn of the absorption edge enters a minimum. This is typical of (intraband) free carrier absorption and known as Moss–Burstein effect in heavily doped semiconductors.^32^ It implies that states at the bottom of the conduction band are occupied (*i.e.* a n-doped semiconductor) and proves the earlier made conjecture that electrons form delocalized bandstates in reduced BaTiO_3_. In contrast, (localized) polaron states would yield shallow traps below the conduction band, and their interband transitions would express themselves as exponential onset of absorption just below the fundamental absorption edge.^33^ Interestingly, a recent report on self-doped TiO_2_ showed that – depending on conditions – this material can be obtained either in the polaron or the bandstate form.^34^ We note that black BaTiO_3_ obtained from Al-reduction displays a very similar UV-VIS spectrum as *e.g.* 1.2H-350.^35^

Fig. 8b shows the Kubelka–Munk (K–M) transformed spectra, which – for comparability across the samples – were normalized. The K–M transformed spectra suggest an increase of the charge carrier concentration within the oxyhydride samples, according to 1.2H-300–1.2H-350–1.2H-400–1.2H-450, that is, the oxyhydride sample with the highest defect concentration possesses the highest concentration, and then decreasing again for 1.2H-500 and 1.2H-600 which are the (core–shell) samples void of H. The trend within the oxyhydride samples is in agreement with the ^1^H MAS NMR spectra when correlating the charge carrier concentration with the Knight shift. The free carrier contributions complicate the estimate of the band gap. Tauc plots – which were not corrected for interband transitions – suggest a band gap of 3.18 eV for BaTiO_3_ and 1.2H-250, and an upward shift to 3.25 eV for 1.2H-300 and 1.2H-350 and to 3.4 eV for 1.2H-450. However, we conjecture that the band gap within these oxyhydride materials is only slightly changed – if at all. The K–M transformed spectra for 1.2H-500 and 1.2H-600 suggest then a significant shift of the absorption edge for the materials void of hydridic H. However, Tauc plot evaluation would not give a reliable determination of the band gap (see ESI, Fig. S13†).

**Fig. 9:**
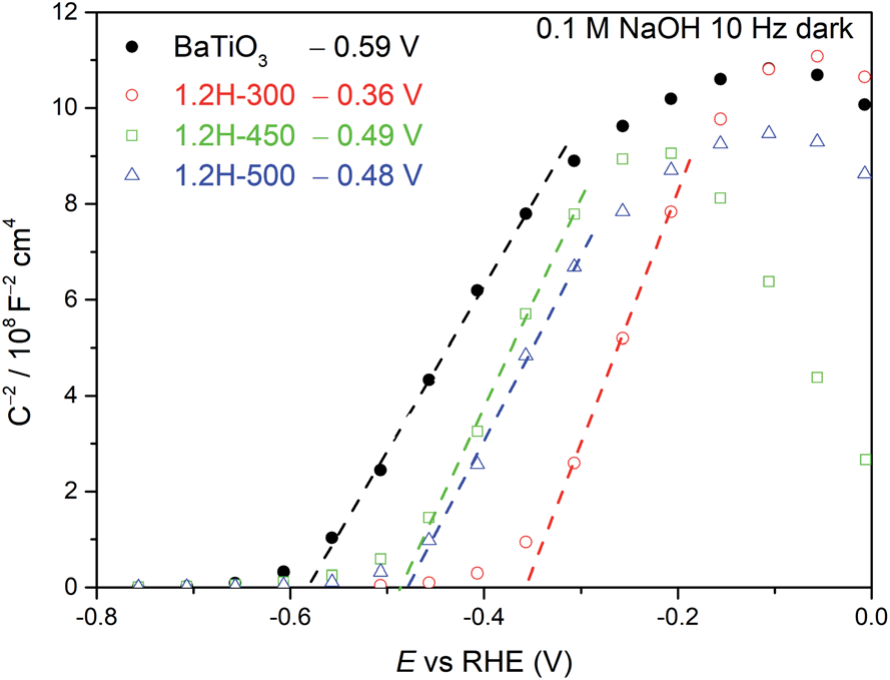
Mott–Schottky plots of BaTiO_3_, 1.2H-300, 1.2H-450, and 1.2H-500 measured at 10 Hz under dark conditions.

Mott–Schottky measurements (Fig. 9) were carried out for 1.2H-300, 1.2H-450, 1.2H-500, and the precursor BaTiO_3_ material. All the reduced samples and the precursor show a positive slope, indicating the semiconducting n-type behavior. The flat band potential of the precursor was estimated to be −0.59 V *vs.* RHE, which is close to previous reported values for BaTiO_3_.^36,37^ In case of a n-type semiconductor, the flat band potential is close to the bottom of the conduction band.^38^ 1.2H-300 (*i.e.* the stoichiometric oxyhydride BaTiO_∼2.9_H_∼0.1_) exhibits a flat band potential at −0.36 V *vs.* RHE, which is a significant positive shift with respect to the pristine BaTiO_3_. For 1.2H-450 and 1.2H-500, corresponding to O-deficient BaTiO_2.6_H_0.1_□_0.3_ and BaTiO_3−*x*_ core/TiO_*x*_(OH)_*y*_ shell material, respectively, the conduction band edge is shifted back to app. −0.5 V *vs.* RHE. Curiously, the slopes remain approximately at the same level for all samples, although the UV-VIS and ^1^H MAS NMR investigations strongly suggest that the reduced samples are metallic and possess different charge carrier concentrations. The slope of a Mott–Schottky plot is inversely proportional to the charge carrier concentration and the dielectric constant. Thus, one may infer that the dielectric constant of the reduced cubic samples is drastically diminished compared to ferroelectric tetragonal BaTiO_3_ (see. *e.g.*ref. 39).

## 4 Conclusions

Hydride reduction has developed into a versatile method for modifying transition metal oxides, yielding O-deficient or mixed anion oxyhydride phases.^1,10,11^ In the case of BaTiO_3_ it appears difficult to control the outcome of hydride reduction and products frequently correspond to highly O-deficient, disordered, cubic phases BaTiO_3−*x*_H_*y*_□_(*x*−*y*)_ with *x* up to 0.6 and *y* in a range 0.04–0.25.^13^ In this work it is shown that the application of LiH as reducing agent allows reduction of BaTiO_3_ at extraordinarily low temperatures, around 300 °C, which affords the oxyhydride BaTiO_∼2.9_H_∼0.1_. Reduction at higher temperatures result in simultaneous O defect formation, BaTiO_2.9−*x*_H_0.1_□_*x*_, and eventually – at temperatures above 450 °C – to samples void of hydridic H. Concomitantly, the particles of samples reduced at high temperatures (500–600 °C) display substantial surface alteration, which is interpreted as the formation of a TiO_*x*_(OH)_*y*_ shell. Thus LiH appears as a versatile reagent to produce distinct forms of reduced, metallic BaTiO_3_.

## Conflicts of interest

There are no conflicts to declare.

## Supplementary Material

RA-010-D0RA07276A-s001
