# Supplementary material for: Trapping of different stages of BaTiO3 reduction with LiH
Source: RSC Adv. 2020 Sep 24;10(58):35356–65. doi: 10.1039/d0ra07276a (PMC9056924; doi:10.1039/d0ra07276a)
Supplement: RA-010-D0RA07276A-s001 [file RA-010-D0RA07276A-s001.pdf]

Electronic Supplementary Material (ESI) for RSC Advances.
This journal is © The Royal Society of Chemistry 2020

SUPPORTING INFORMATION

Trapping different stages of $BaTiO_3$ reduction with LiH

Hua Guo,[1] Aleksander Jaworski,[1] Zili Ma,[1,2] Adam Slabon,[1] Zoltan Bacsik,[1] Reji Nedumkandathil,[1] and Ulrich Häussermann[1,*]

[1]*Department of Materials and Environmental Chemistry, Stockholm University, SE-10691 Stockholm, Sweden*
[2]*Institute of Inorganic Chemistry, RWTH Aachen University, Landoltweg 1, DE-52074 Aachen, Germany*

Refinement details and Rietveld plots for the products from LiH reduction

Refinement results summary and Rietveld plots

SEM analysis for 1.2H-700

PXRD and SEM characterization of products after TG/air treatment

^{7}Li-NMR MAS spectra for 1.2H-500, 3H-500, 10H-500, 1.2H-600, 3H-600.

Results of WDX analysis for $BaTiO_3$, 1.2H-500, and 1.2H-600

Details of XRF investigation

Compilation of 3H and 10H results

Tauc plots

Refinement details and Rietveld plots for the products from LiH reduction

Table S1. Refinement results summary

Sample	Lattice parameters (Å)	Volume (Å^3)	χ^2	R_{BRAGG}	R_F
1.2H-250	a = 3.9955 (1)<br>c = 4.0247 (2)	64.250 (4)	3.89	4.86	3.31
1.2H-300	4.0046 (1)	64.219 (5)	4.75	8.85	6.98
1.2H-350	4.0069 (2)	64.329 (5)	4.09	11.3	9.04
1.2H-375	4.0084 (1)	64.405 (4)	3.39	5.91	3.20
1.2H-400	4.0122 (1)	64.588 (3)	1.25	9.44	7.50
1.2H-425	4.0156 (1)	64.754 (3)	1.82	4.60	3.04
1.2H-450	4.0212 (1)	65.021 (3)	1.45	8.63	8.93
1.2H-500	4.0212 (1)	65.022 (4)	3.26	4.47	2.72
1.2H-600	4.0200 (1)	64.965 (4)	2.74	5.37	3.45
3H-350	4.0090 (1)	64.433 (3)	3.74	4.25	2.36
3H-500	4.0314 (1)	65.520 (3)	4.71	5.45	3.60
10H-350	4.0107 (1)	64.516 (3)	6.35	4.22	2.79
10H-500	4.0338 (1)	65.636 (3)	3.75	6.51	3.97

Compilation of Rietveld plots of products from reductions using 1.2 M, 3 M, and 10 M LiH.

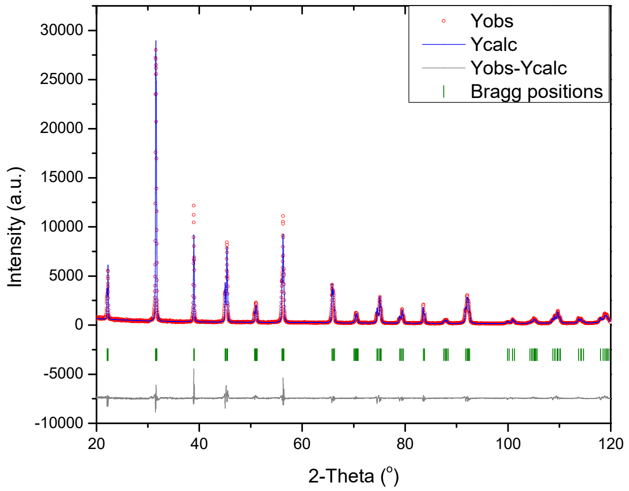

Figure S1a. Refinement of 1.2LiH-250.

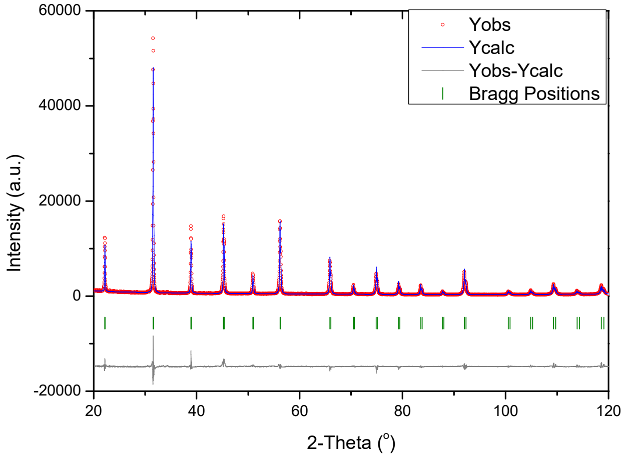

Figure S1b. Refinement of 1.2LiH-300.

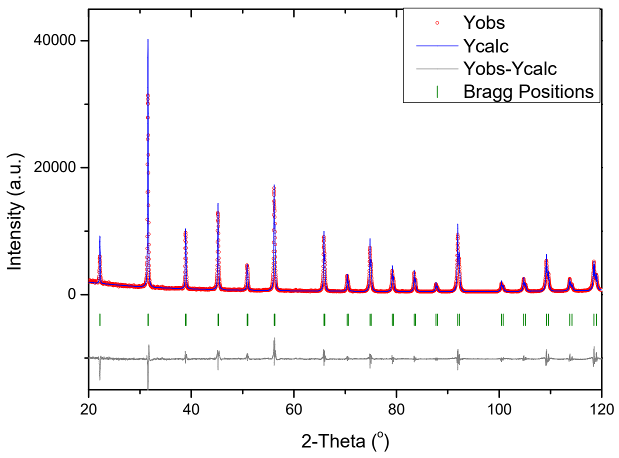

Figure S1c. Refinement of 1.2LiH-350

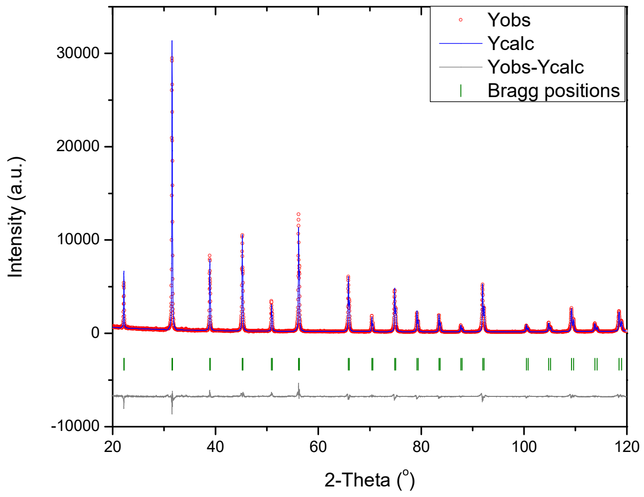

Figure S1d. Refinement of 1.2LiH-375.

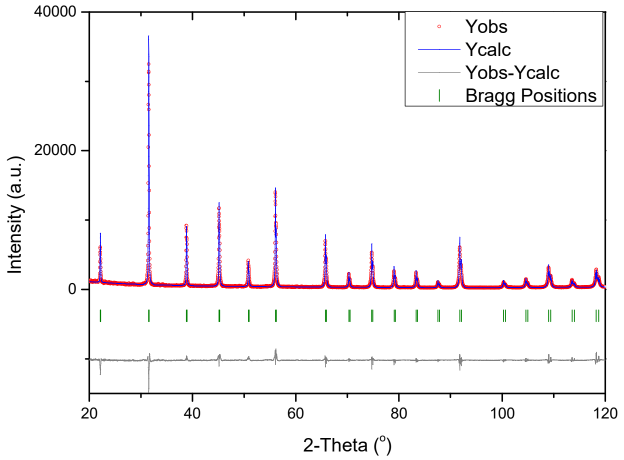

Figure S1e. Refinement of 1.2LiH-400.

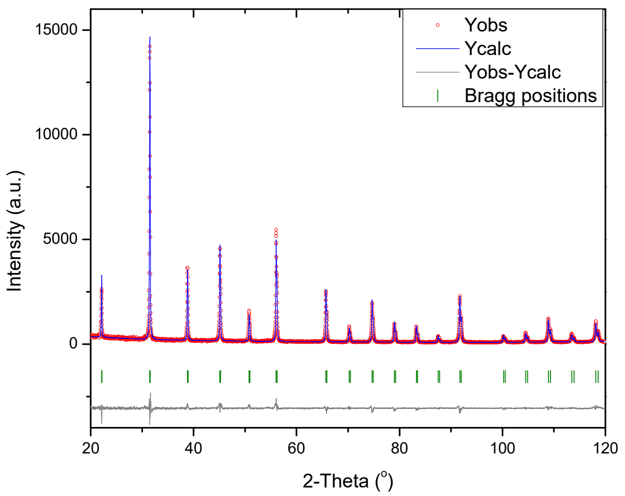

Figure S1f. Refinement of 1.2LiH-425.

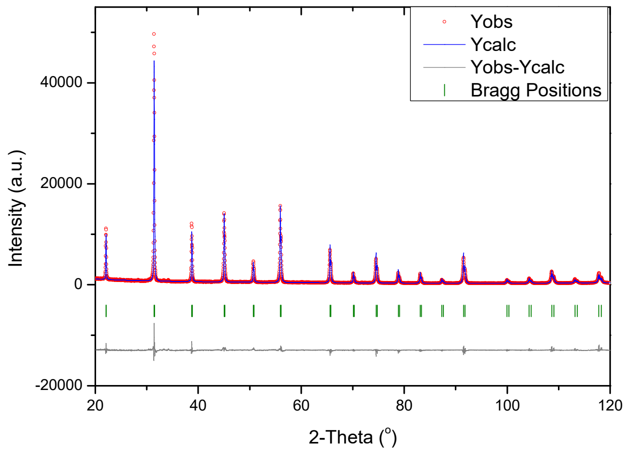

Figure S1g. Refinement of 1.2LiH-450.

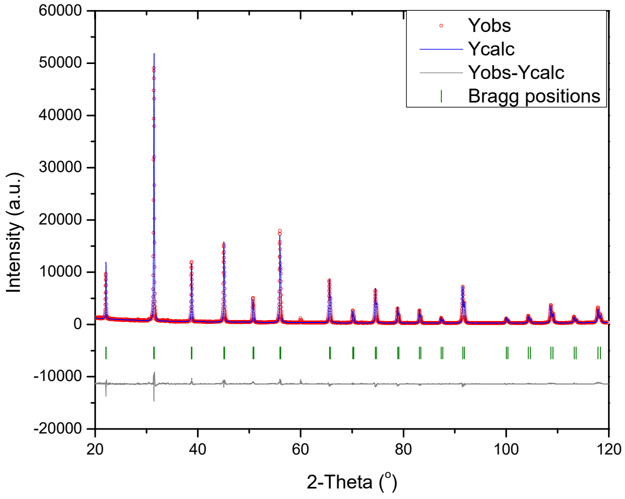

Figure S1h. Refinement of 1.2LiH-500.

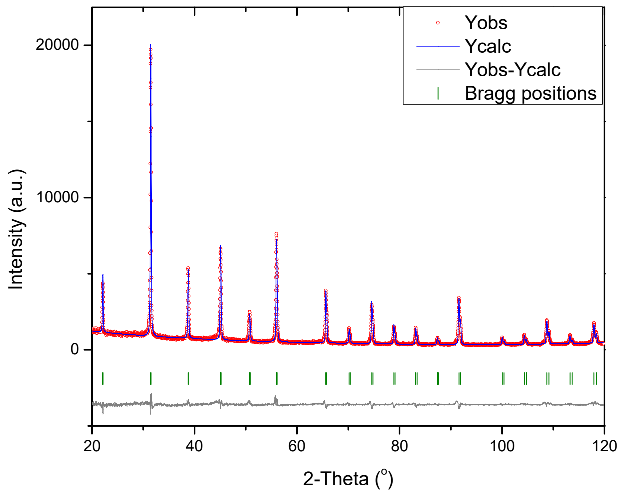

Figure S1i. Refinement of 1.2LiH-600.

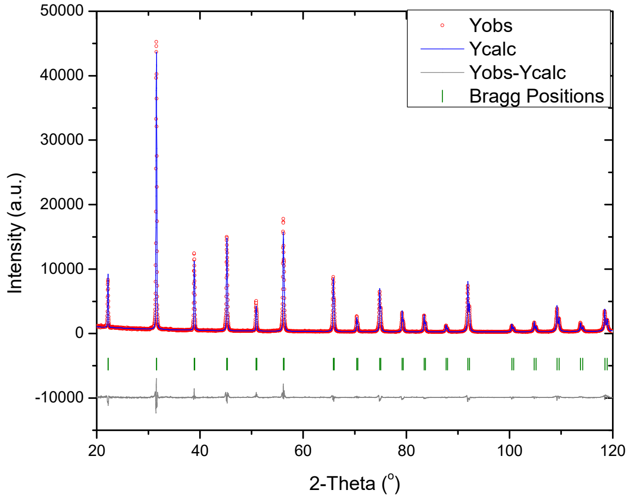

Figure S2a. Refinement of 3LiH-350

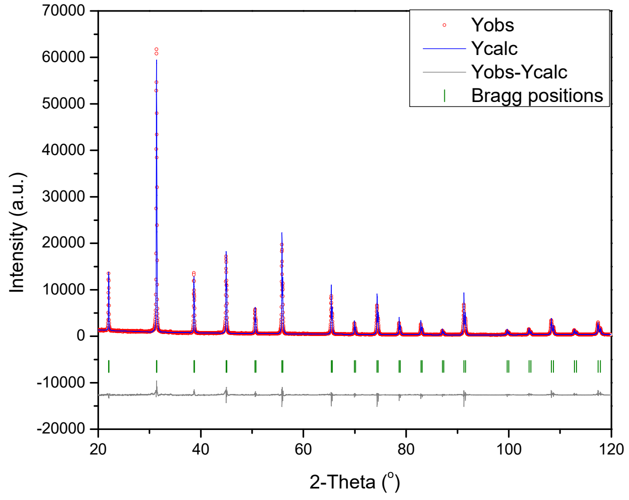

Figure S2b. Refinement of 3LiH-500.

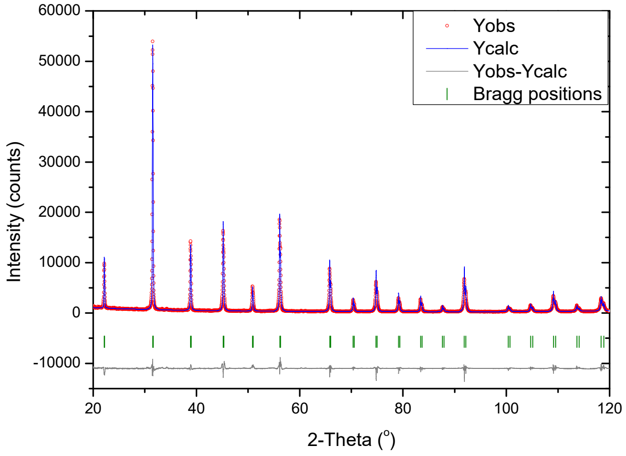

Figure S3a. Refinement of 10LiH-350.

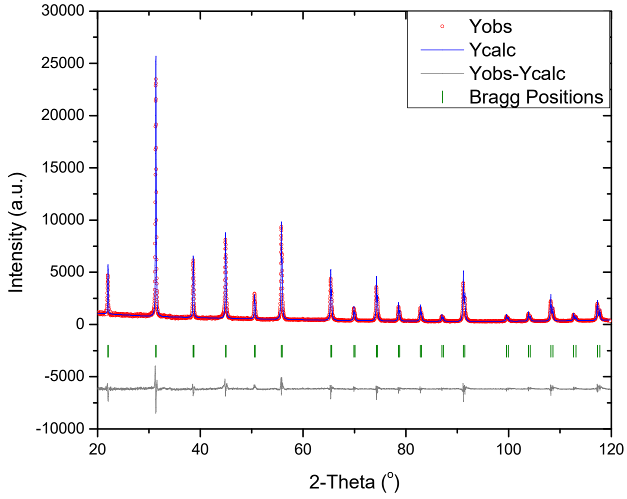

Figure S3b. Refinement of 10LiH-500.

SEM characterization of 1.2H-700

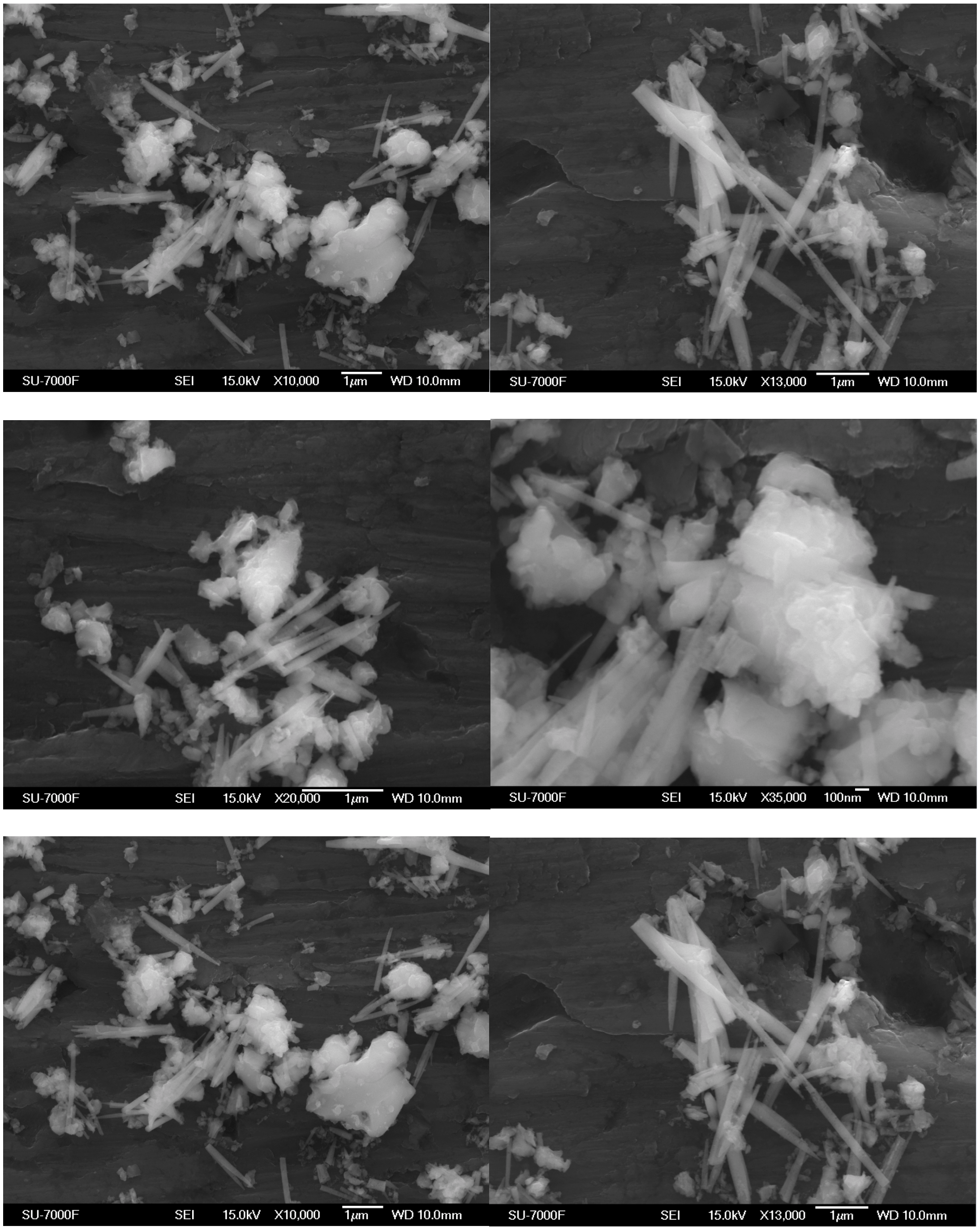

Figure S4. SEM images of 1.2 M LiH reduced sample at 700 °C.

Characterization of samples after TG/air treatment

PXRD

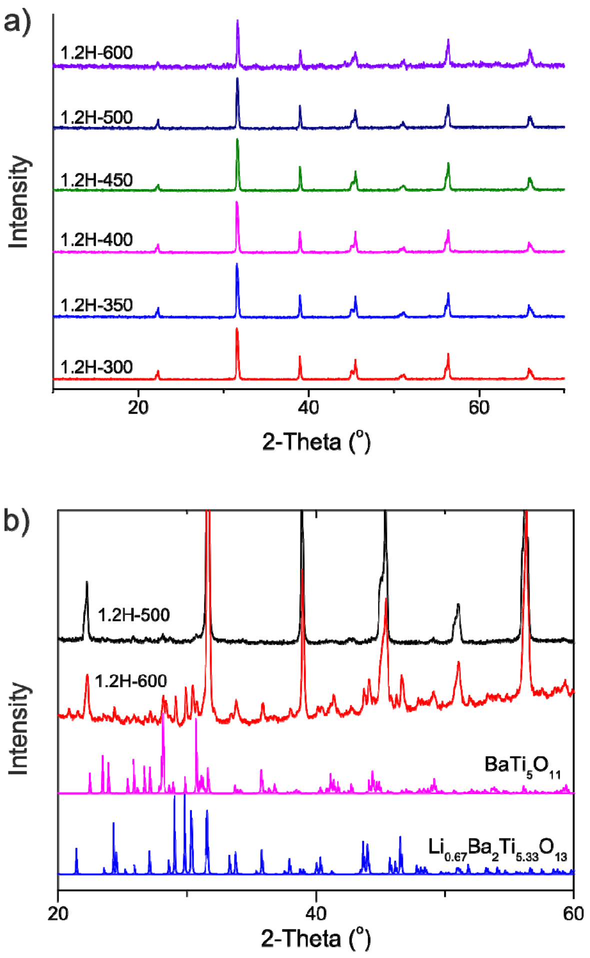

Figure S5. a) PXRD patterns of 1.2 M LiH reduced samples after TG/air treatment to 900 °C. b) Close up of 1.2H-500 and 1.2H-600 patterns showing the presence of secondary phases. The calculated patterns for $BaTi_5O_{11}$ (monoclinic, P21/c, COD code 96-210-7201) and $Li_{0.67}Ba_2Ti_{5.33}O_{13}$ (monoclinic, C2/m, COD code 04-012-6914) included for comparison.

SEM

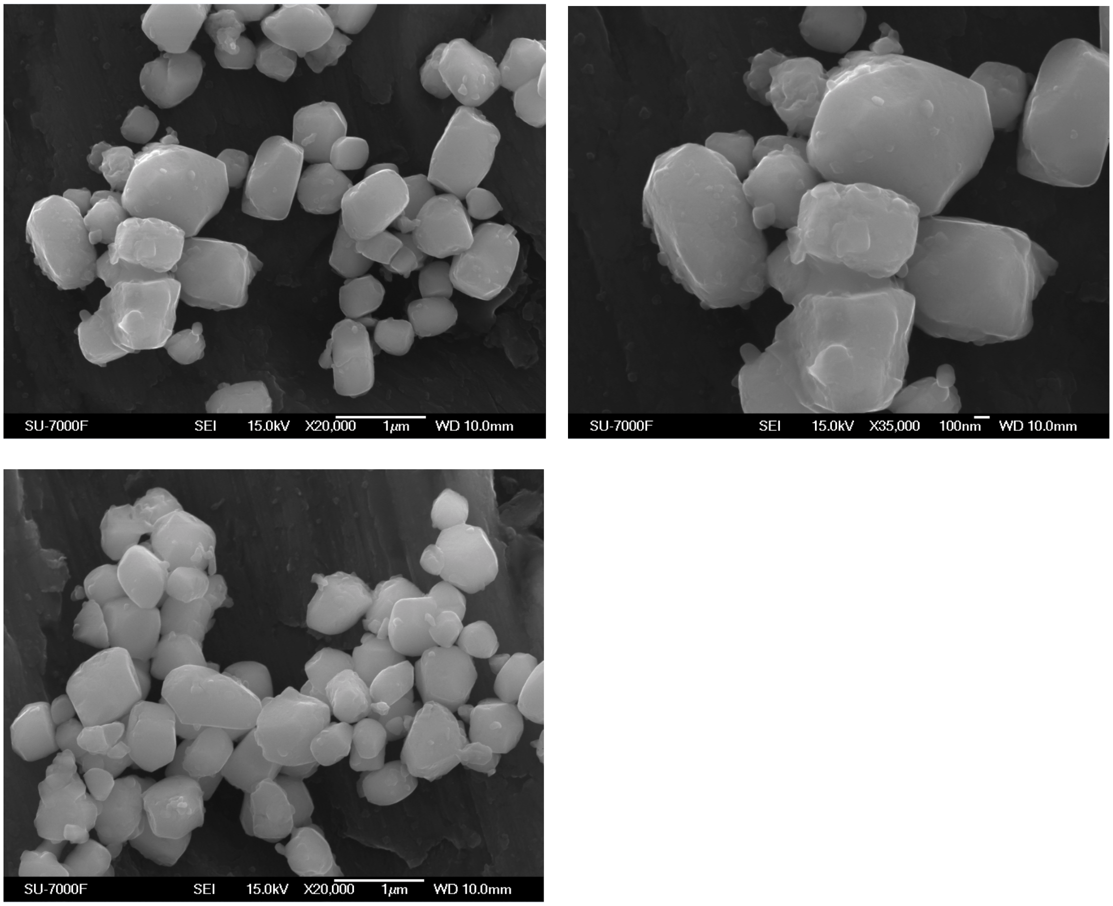

Figure S6a. SEM images of 1.2-450 after TG/air treatment to 900 °C.

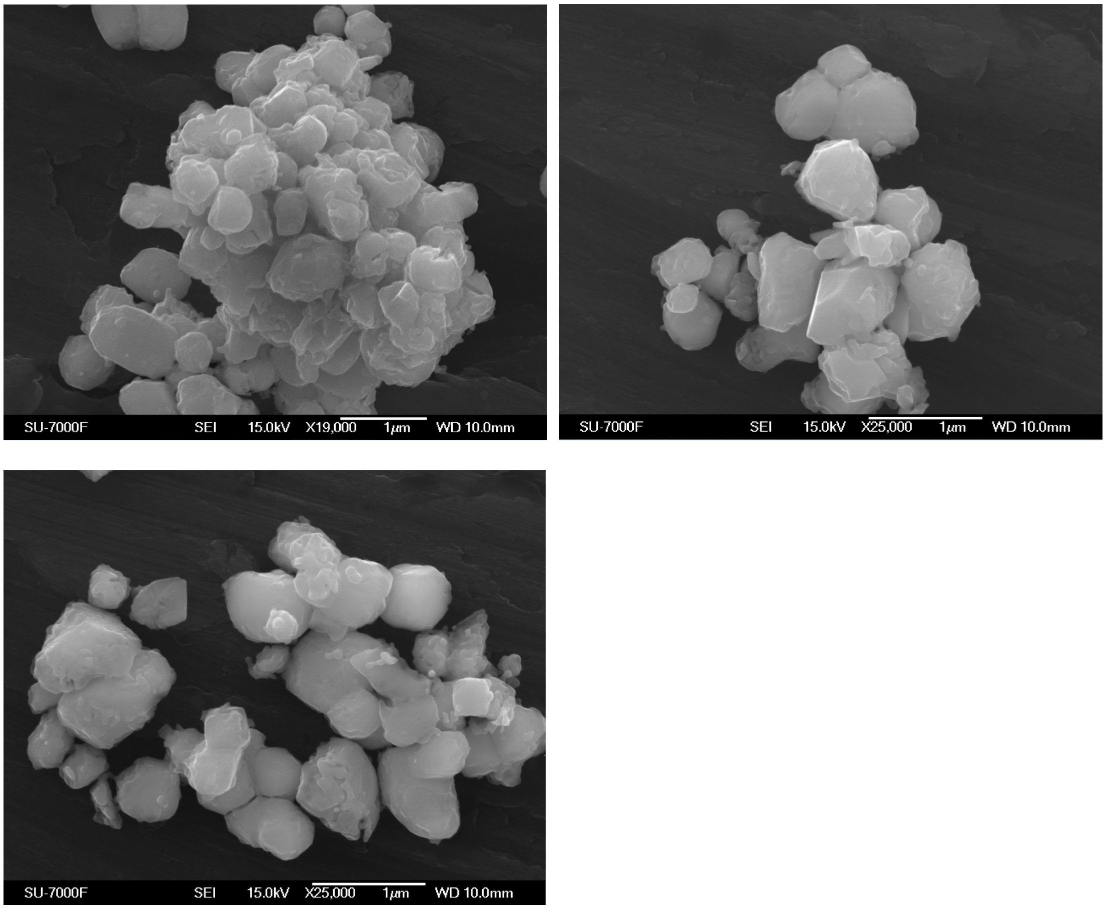

Figure S6b. SEM images of 1.2-500 after TG/air treatment to 900 °C.

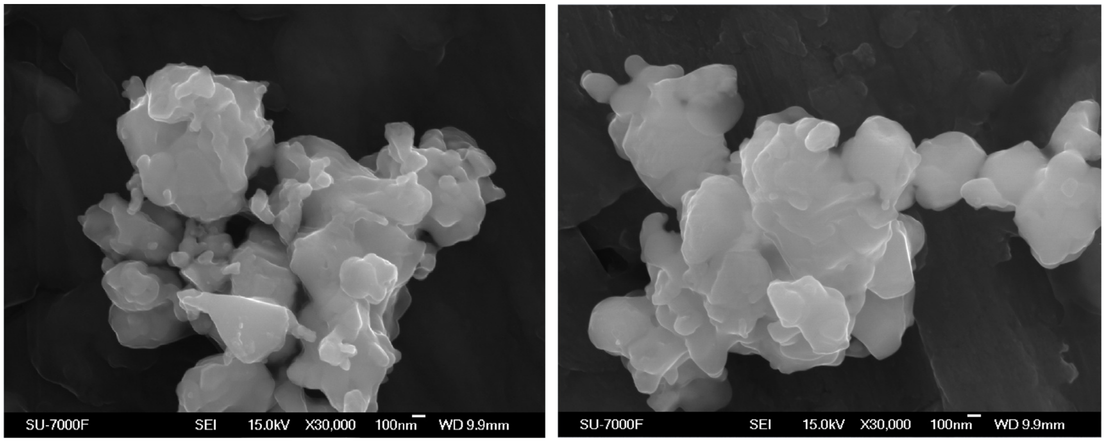

Figure S6c. SEM images of 1.2-600 after TG/air treatment to 900 °C.

^{7}Li MAS NMR

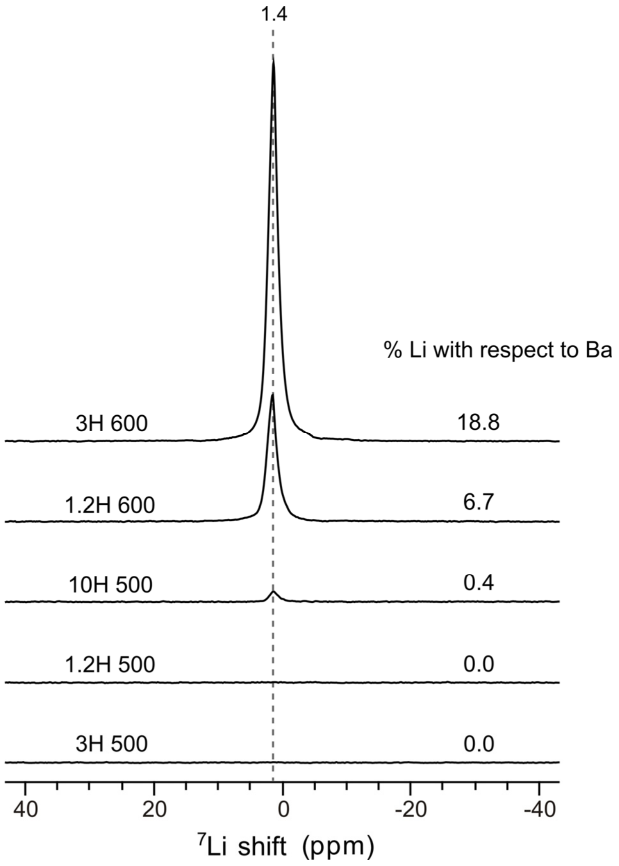

Figure S7. ^{7}Li MAS NMR spectra of products obtained from various LiH reductions during 48 h at 500 and 600 °C.

Details of XRF investigation

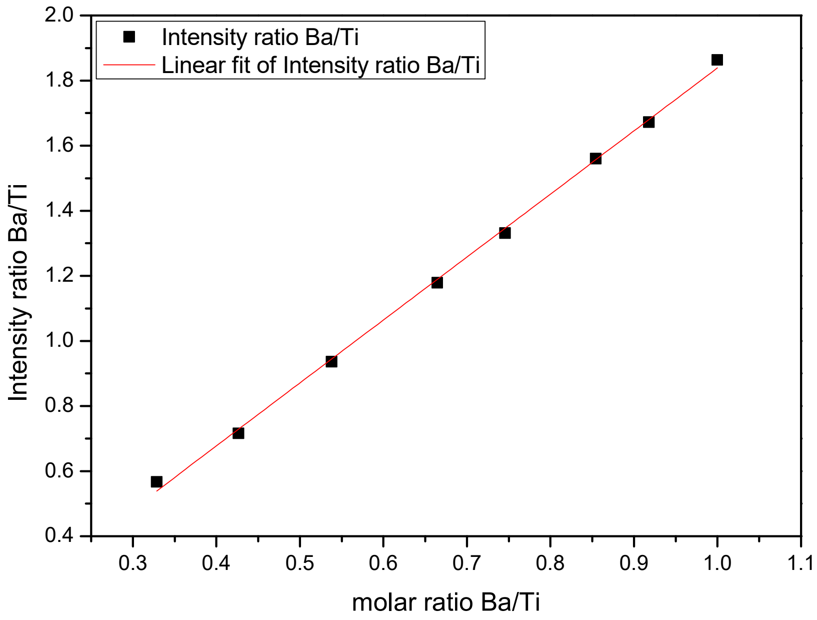

S8. XRF calibration of Ba/Ti molar ratio using $BaTiO_3$/TiO_2 sample mixtures as standards. The molar ratio Ba/Ti of reduced $BaTiO_3$ samples was then extracted from this calibration.

Table S2. XRF quantification results of standards.

Molar ratio Ba/Ti	Measured intensity ratio Ba/Ti
1.000	1.86
0.918	1.67
0.854	1.56
0.746	1.33
0.665	1.18
0.538	0.936
0.427	0.716

Table S3. Summary XRF quantification of $BaTiO_3$-LiH reduced samples.

sample	Measured intensity ratio Ba/Ti	Molar ratio Ba/Ti
1.2H_350	1.826	0.993
1.2H_400	1.804	0.982
1.2H_450	1.699	0.928
1.2H_500	1.487	0.818
1.2H_600	1.062	0.598
1.2H_700	0.633	0.377
3H_350	1.800	0.980
3H_500	0.973	0.553

10H_350	1.800	0.980
10H_500	0.876	0.502

We considered the matrix effect (that might occur in the presence of lithium and different concentration of oxygen) by testing a standard mixture with a Ba:Ti molar ratio of 0.67:1 to which different amounts of Li_2CO_3 were added (Li:Ti molar ratios of 0.17:1 and 0.33:1). The results showed that calculated results were well within testing error, see Table S4 below.

Table S4. Ba:Ti molar ratios from XRF testing with added Li_2CO_3.

Molar ratio Ba/Ti	Molar ratio Li/Ti	Intensity ratio Ba/Ti	Difference
0.665	0	1.179	0
0.665	0.167	1.188	+0.7%
0.665	0.333	1.204	+2.2%

WDX results

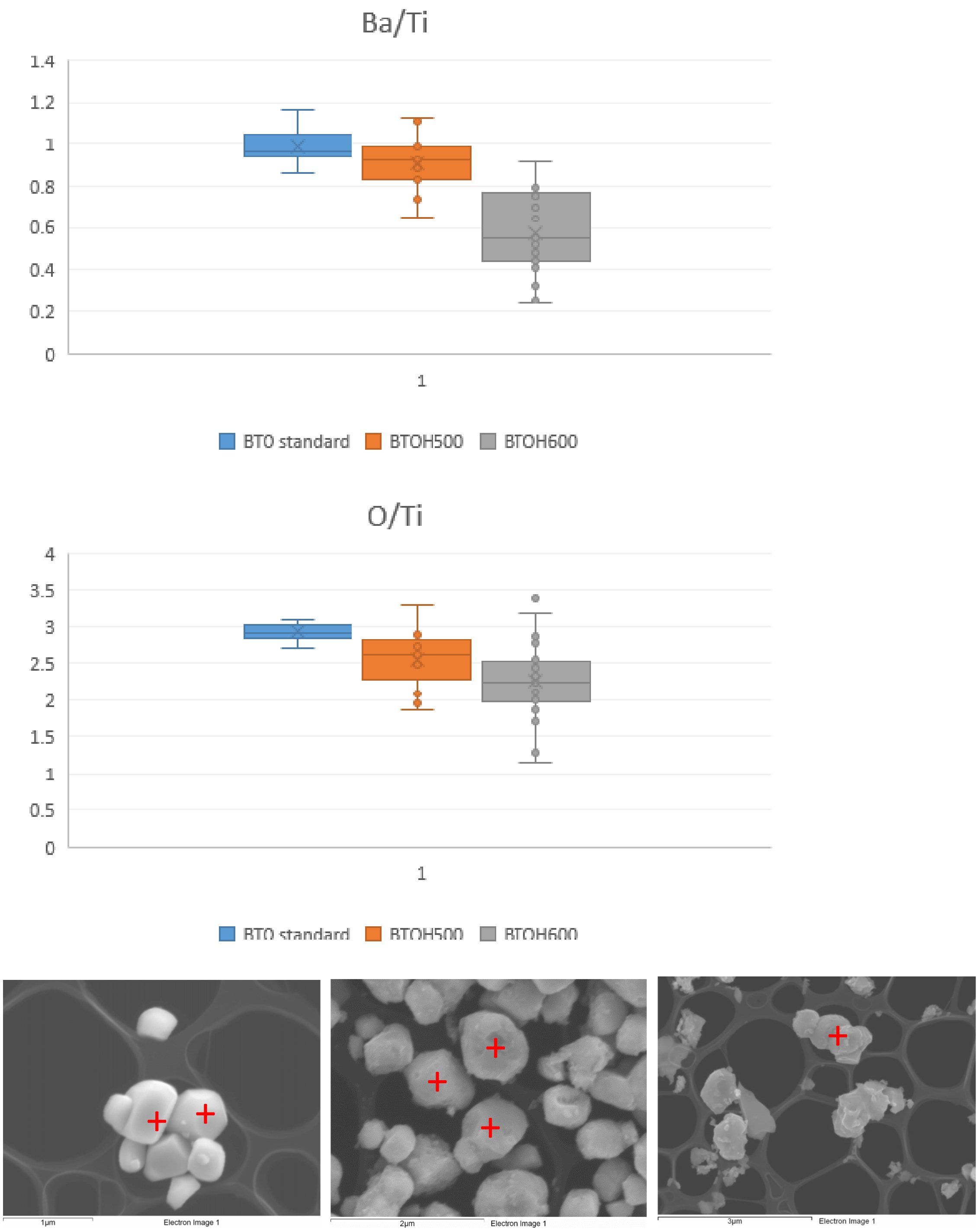

Precursor 1.2H-500 1.2H-600

Figure S9. WDX analysis of the precursor and the reduced samples 1.2H-500 and 1.2H-600. The graphs summarize measured Ba/Ti and O/Ti ratios. Note that data scatter rather widely, but there is a clear trend in the changed ratios.

Compilation of results from reductions using 3 M and 10 M LiH

PXRD

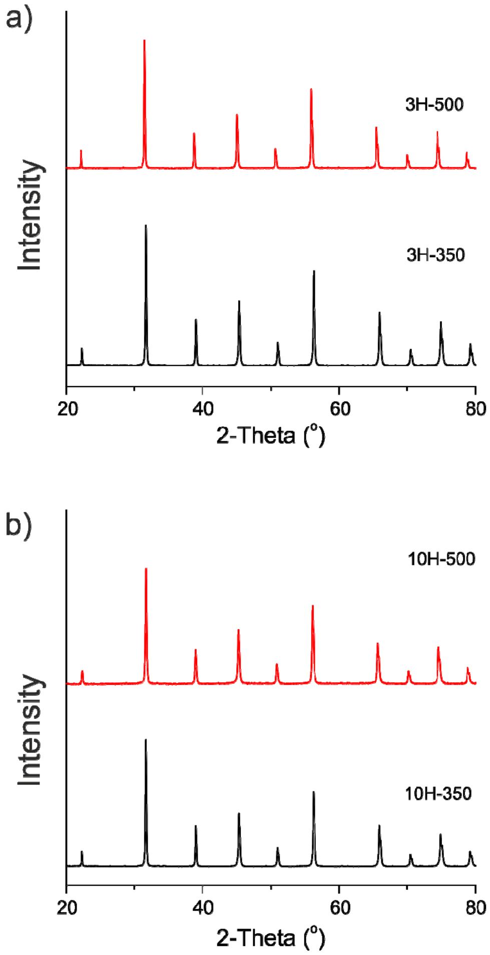

S10. PXRD patterns of 3M LiH (a) and 10M LiH (b) reduced samples at 350 and 500 °C.

SEM

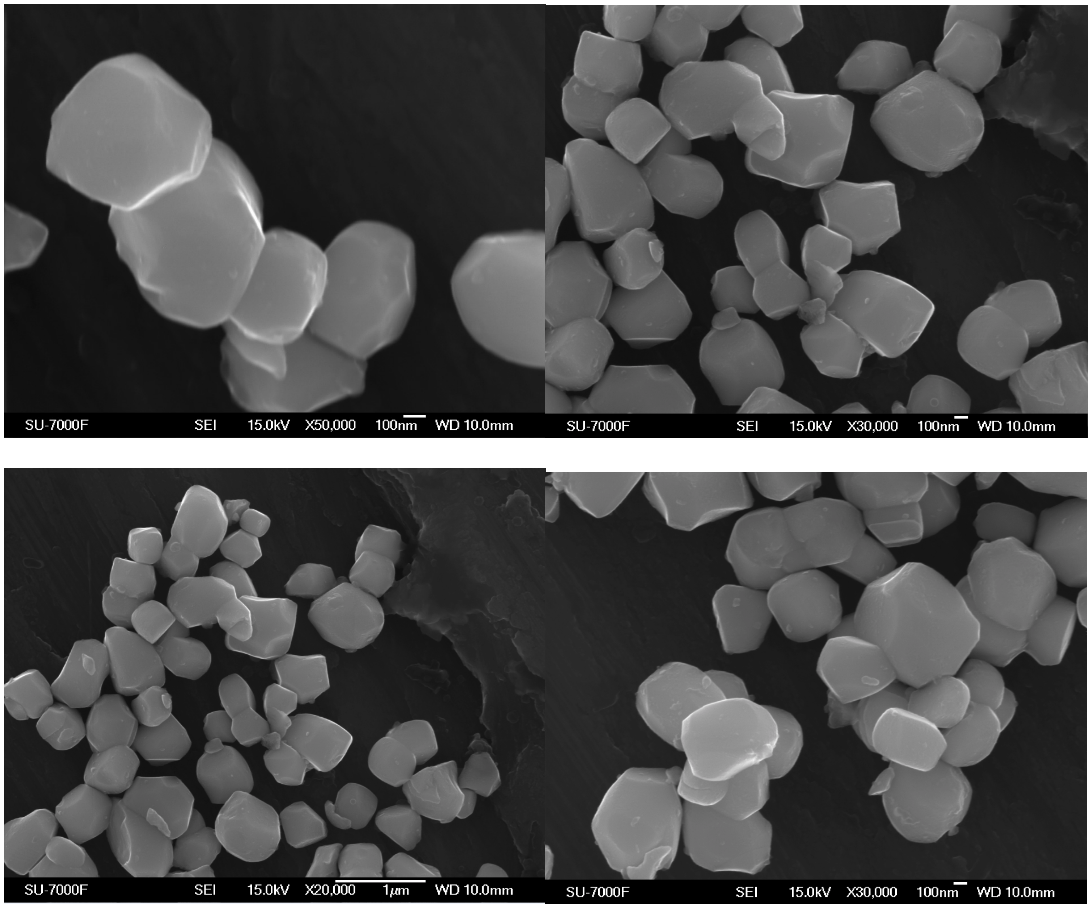

Figure S11a. SEM images of 3 M LiH reduced sample at 350 °C.

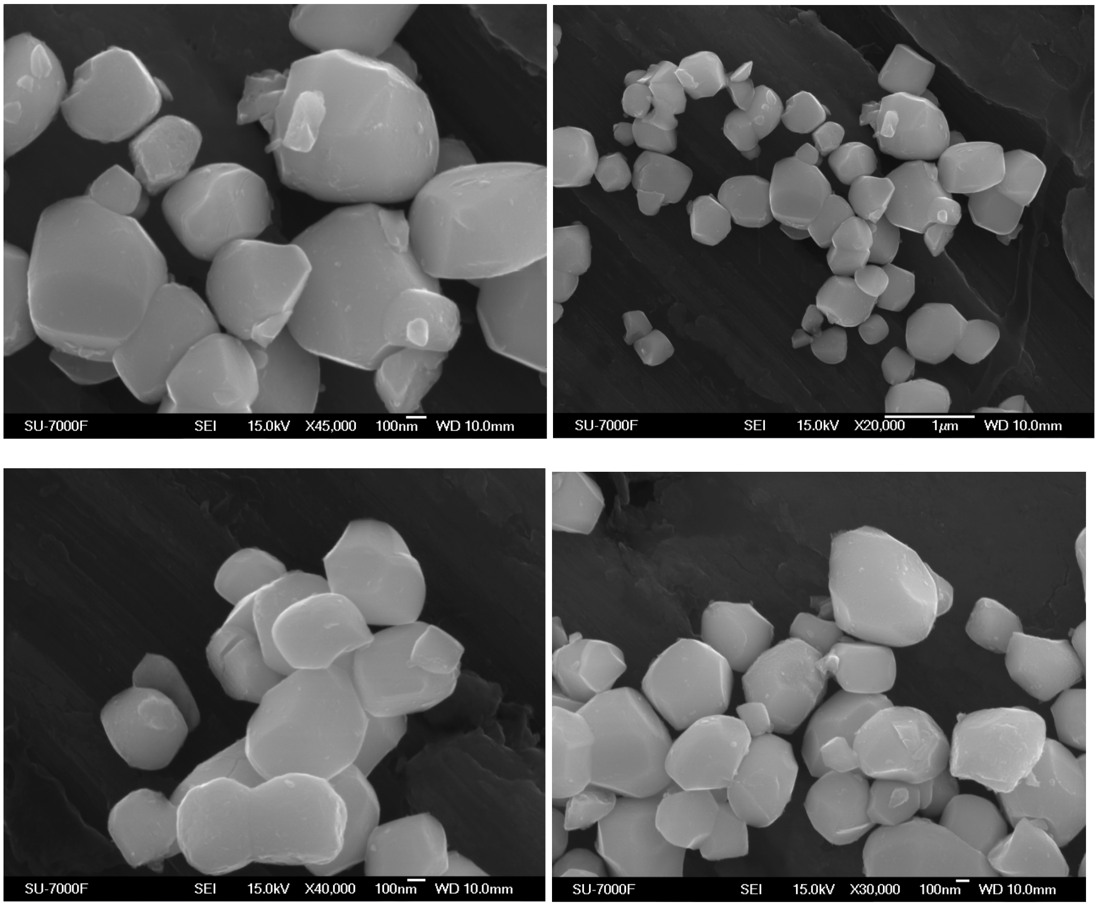

Figure S11b. SEM images of 10 M LiH reduced sample at 350 °C.

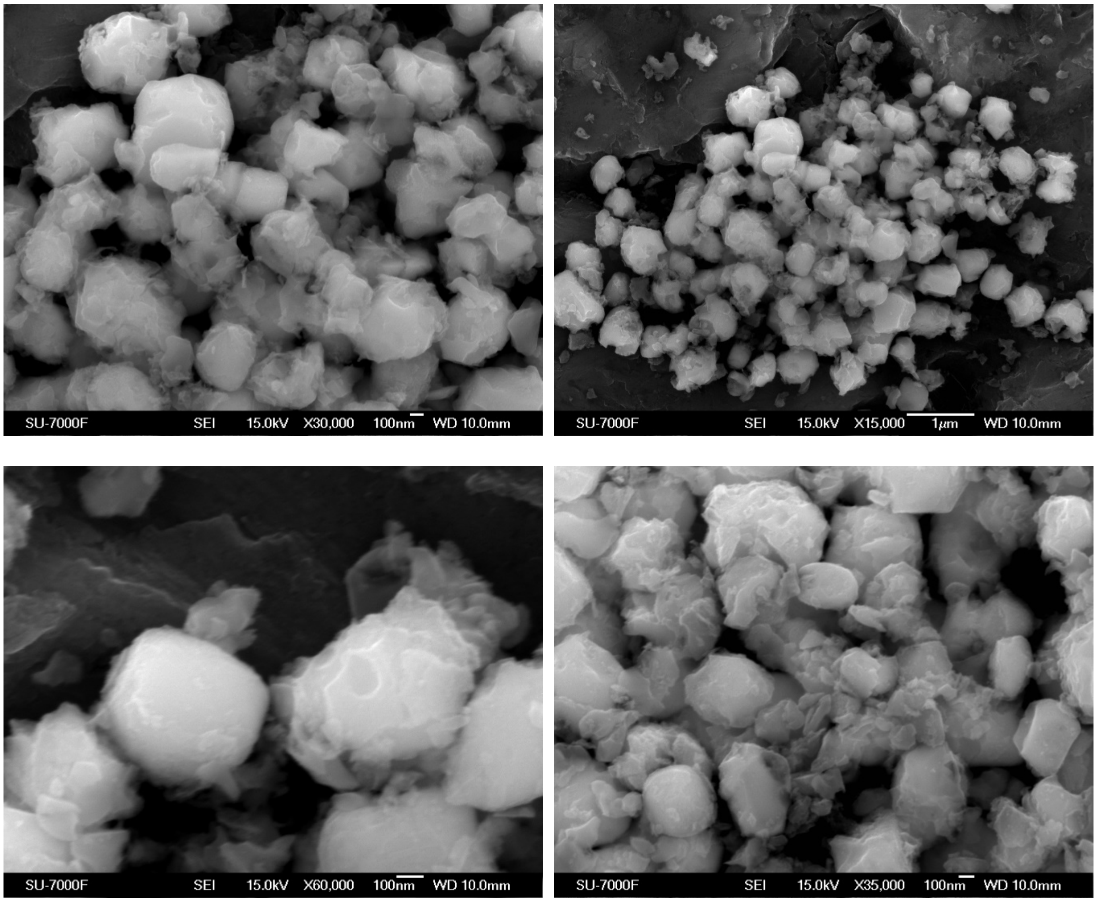

Figure S11c. SEM images of 3 M LiH reduced sample at 500 °C.

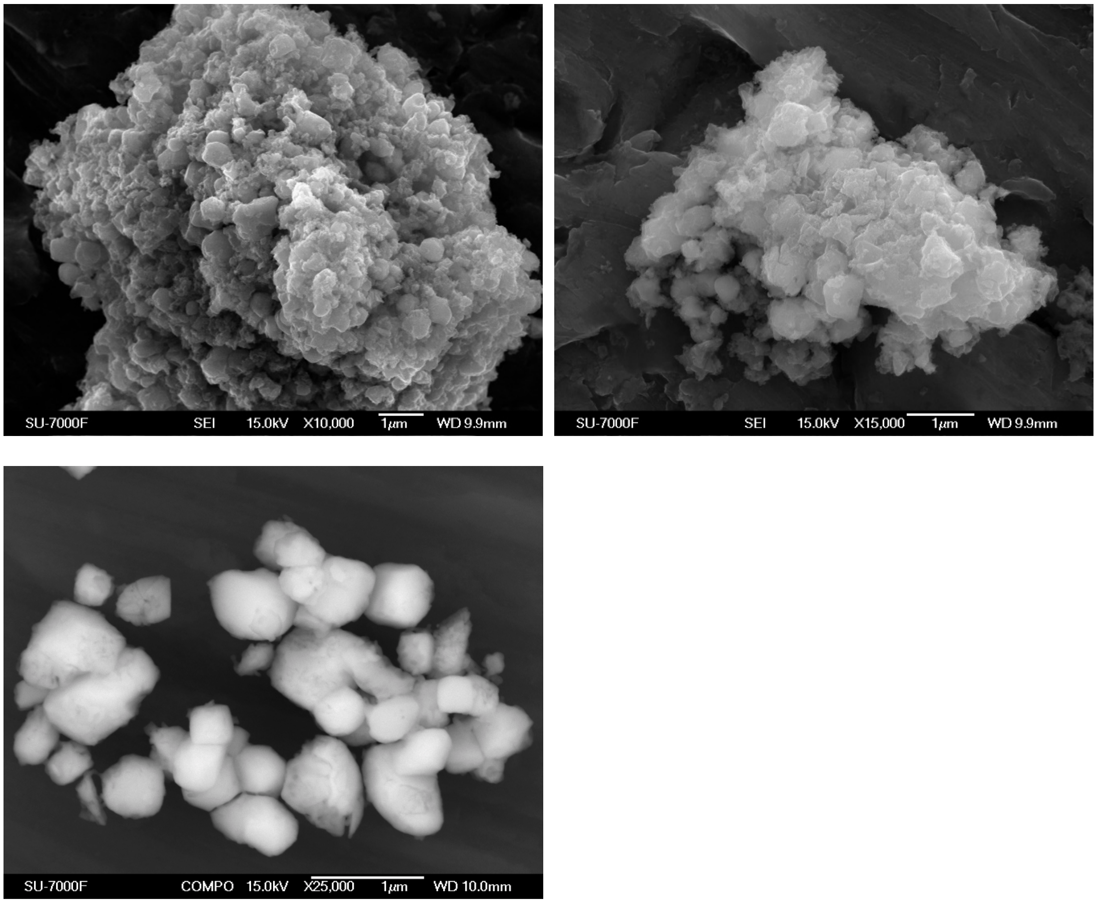

Figure S11d. SEM images of 10 M LiH reduced sample at 500 °C.

PXRD after TG

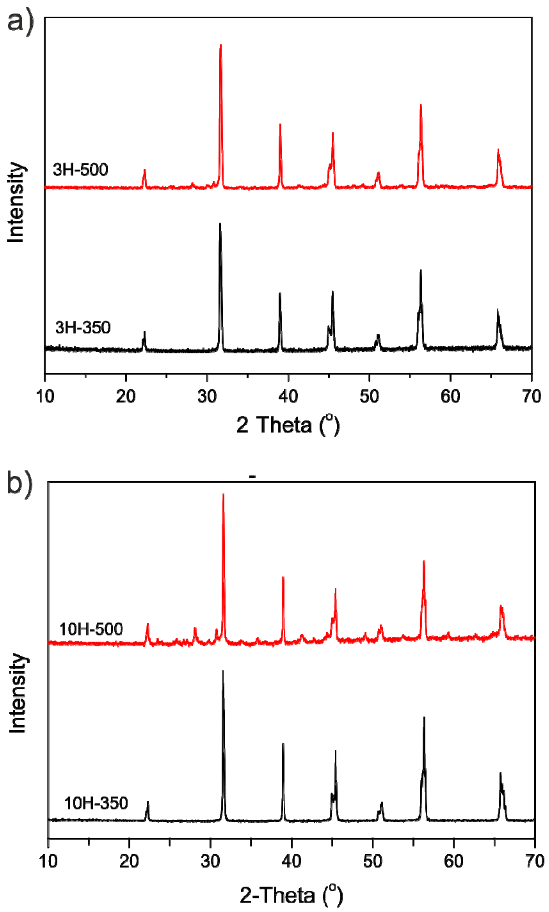

Figure S12. PXRD patterns of 3 M LiH (a) and 10 M LiH (b) reduced samples after TG/air treatment to 900 °C.

Tauc plots

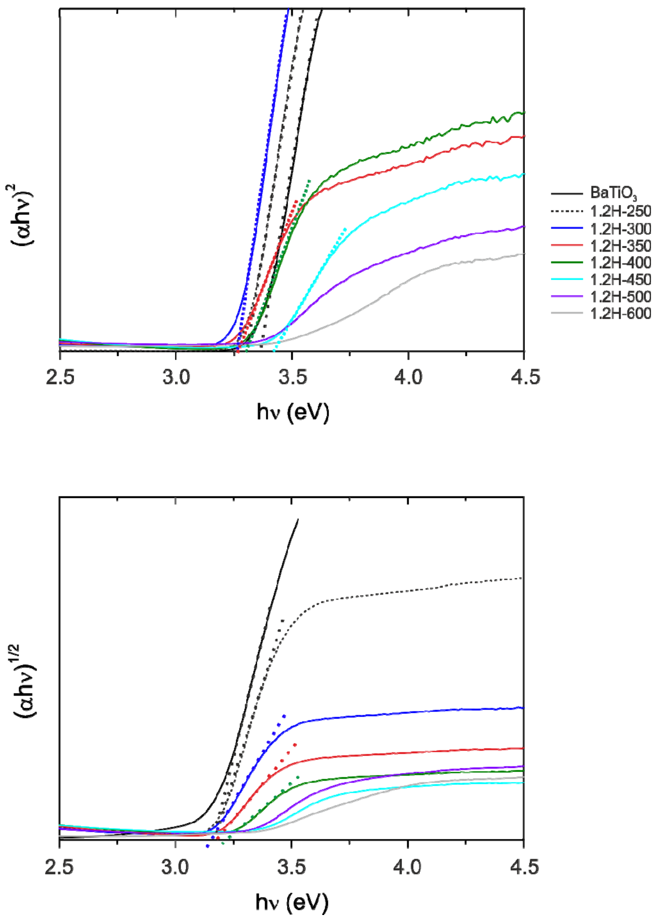

Figure S13. Tauc plots for direct (upper figure) and indirect (lower figure) band gap evaluation of $BaTiO_3$ precursor and reduced samples.
